# Comparing and Quantifying the Efficiency of Cocrystal Screening Methods for Praziquantel

**DOI:** 10.1021/acs.cgd.2c00615

**Published:** 2022-08-25

**Authors:** Maxime D. Charpentier, Jan-Joris Devogelaer, Arnoud Tijink, Hugo Meekes, Paul Tinnemans, Elias Vlieg, René de Gelder, Karen Johnston, Joop H. ter Horst

**Affiliations:** † EPSRC Centre for Innovative Manufacturing in Continuous Manufacturing and Crystallization (CMAC), University of Strathclyde, Technology and Innovation Centre, 99 George Street, Glasgow G1 1RD, U.K..; ‡ Institute for Molecules and Materials, Radboud University, Heyendaalseweg 135, 6525AJ Nijmegen, The Netherlands; § Department of Chemical and Process Engineering, University of Strathclyde, James Weir Building, 75 Montrose Street, Glasgow G1 1XJ, U.K.; ∥ Laboratoire Sciences et Méthodes Séparatives, Université de Rouen Normandie, Place Emile Blondel, 76821 Mont Saint Aignan Cedex, France

## Abstract

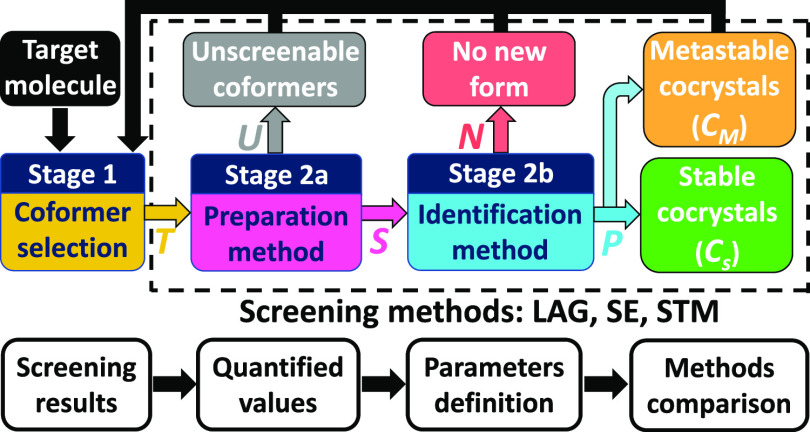

Pharmaceutical cocrystals are highly interesting due to their effect on physicochemical properties and their role in separation technologies, particularly for chiral molecules. Detection of new cocrystals is a challenge, and robust screening methods are required. As numerous techniques exist that differ in their crystallization mechanisms, their efficiencies depend on the coformers investigated. The most important parameters characterizing the methods are the (a) screenable coformer fraction, (b) coformer success rate, (c) ability to give several cocrystals per successful coformer, (d) identification of new stable phases, and (e) experimental convenience. Based on these parameters, we compare and quantify the performance of three methods: liquid-assisted grinding, solvent evaporation, and saturation temperature measurements of mixtures. These methods were used to screen 30 molecules, predicted by a network-based link prediction algorithm (described in Cryst. Growth Des. **2021,***21*(6), 3428–3437) as potential coformers for the target molecule praziquantel. The solvent evaporation method presented more drawbacks than advantages, liquid-assisted grinding emerged as the most successful and the quickest, while saturation temperature measurements provided equally good results in a slower route yielding additional solubility information relevant for future screenings, single-crystal growth, and cocrystal production processes. Seventeen cocrystals were found, with 14 showing stability and 12 structures resolved.

## Abstract 2

Detection of new cocrystals is key for the pharmaceutical industry, and efficient screening methods are required. The results of a cocrystal screening campaign for praziquantel are studied to compare thoroughly the advantages and drawbacks of three screening methods used: liquid-assisted grinding, solvent evaporation, and saturation temperature measurements. With the definition of quantified values and the calculation of comparison parameters, it is possible to compare quantitatively the efficiencies of screening methods.

## 1 Introduction

Multicomponent crystal classes vary with the nature of the components sharing the structure and include salts consisting of ions, solvates when one or more of the components is a solvent, or cocrystals when nonsolvent neutral coformers associate as supramolecular synthons.^1−4^ For structures containing more than two components, combined subclasses may also exist, for instance, cocrystal solvates.^2^ Screening for multicomponent crystals, and especially cocrystals, is of strong interest to the pharmaceutical industry as it is a route toward optimization of drug physicochemical properties, such as solubility, bioavailability, mechanical/humidity/thermal stability, and compressibility,^5−11^ without modifying their medical action, and can also be used as a separation technology.^12^ When active pharmaceutical ingredients (APIs) are chiral, discovering solid forms can also prompt new chiral separation possibilities.^13−16^ The marketing of enantiopure drugs is an essential topic because racemic mixtures, that is, equimolar ratio of enantiomers, contain only half of the active form, the other half being the opposite-enantiomer impurity, which, besides bringing economical constraints,^17^ can also produce unwanted side effects.^18−20^ As 90–95% of chiral systems synthetized as racemic mixtures crystallize as racemic compounds, that is, crystal structures containing both enantiomers, their chiral resolution is tricky or even impossible.^21^ Introducing only achiral coformers to a racemic compound system can generate multicomponent crystals that can either be racemic or be a conglomerate of enantiopure crystals.^22,23^ For conglomerates, chiral resolution processes are then possible such as preferential crystallization, temperature-cycling deracemization, or Viedma ripening.^24−34^ On introducing a chiral coformer, a dissymmetry is induced when forming multicomponent crystals, and outcomes can be either diastereomeric pairs of enantiopure phases or enantiospecific systems, that is, only one enantiomer forms a new multicomponent crystal. Both outcomes are favorable for chiral resolution.^13−15,35^

**Figure 1:**
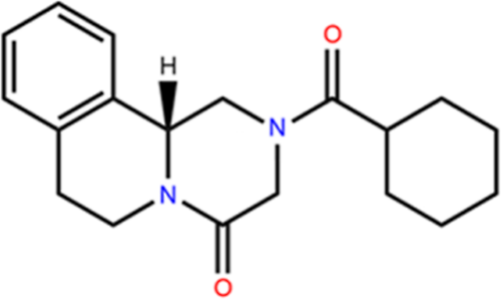
Molecular structure of Praziquantel (PZQ).

Praziquantel (PZQ) (shown in Figure 1), the model chiral compound of this study, presents several challenges that could be solved by multicomponent crystal formation. PZQ is the standard medicine for a parasitic worm infection named schistosomiasis causing the death of about 280,000 people annually in underdeveloped regions of Africa, South America, and Asia.^36−38^ Searches for multicomponent crystals are performed either to improve the drug physicochemical properties^39−42^ or to separate enantiomers with the purpose to produce an enantiopure drug.^43^ Indeed, chiral resolution strategies are sought for PZQ,^43−45^ currently marketed as a racemic compound, as only its *R*-enantiomer possesses the desired pharmaceutical action, whereas the *S*-enantiomer causes side effects such as a bitter taste. Moreover, the presence of the undesired enantiomer means a higher overall dosage is required that is problematic for young children, and chiral separation would lower this dosage. Screening of new multicomponent crystals is then necessary to find systems allowing enantiomeric resolution.

As PZQ does not possess ionizable functions, salt formation strategy is excluded, while cocrystallization and solvate formation are always possible for organic molecules as these mechanisms involve intermolecular interactions like hydrogen bonds.^46,47^ Cocrystals are generally preferred as they are more stable than solvates with temperature and have a larger accessible pool of compounds “Generally Recognized As Safe” (GRAS compounds) for coformers than solvate-formers,^48^ although pure APIs can be more easily separated from solvates than from cocrystals.^49^ Cocrystallization is, therefore, a topic of interest within the pharmaceutical industry in recent years with the emergence of many cocrystal preparation methods, which can involve transformations in the solid state induced by energy sources that can be mechanical (grinding,^50^ cryomilling, and high-shear granulation), thermal (thermal treatment, crystallization from the melt, and hot-melt extrusion), or based on sound/ultrasound, microwaves, or electrical current.^51,52^ Cocrystallization can be mediated by the presence of solvents, stirring slurries to induce a phase transition, cooling/evaporating/adding an antisolvent to undersaturated solutions, or using supercritical fluids, spray-drying, and freeze-drying technologies.^52,53^ All these methods present advantages and disadvantages, with alternative paths to cocrystal synthesis and experimental limitations that vary with the nature of the coformers. Indeed, the molar ratio between the coformers used will differ with the method, as well as the nature and amount of energy applied. Some techniques can also be nonapplicable to certain coformers that can for instance present thermal or mechanical degradation, reactions with a component/solvent, or formation of amorphous material or unwanted phases. No cocrystallization technique has proven to be universal, but the choice of methods used for detection of cocrystal formation can be optimized.

A typical screening technique is liquid-assisted grinding (LAG),^54−58^ which is a mechanochemical method that is based on absorption of kinetic energy to enable cocrystallization. Here, the components are ground manually or with a ball mill. Potential cocrystallization is enhanced with a small amount of solvent added as a catalyst to assist the transformation process. Solvent evaporation (SE)^55,56,59^ is also commonly used and relies on the evaporation of a small volume of initially undersaturated solution with a volatile solvent. The evaporation gradually concentrates the compositions to drive cocrystallization. Another solvent-based screening method, that we name STM, uses saturation temperature measurements of coformer mixtures obtained via cooling crystallization.^60−63^ Saturation temperatures, that is, solubilities, of coformers are measured separately and then for mixtures with compositions chosen as a function of pure coformer solubilities. A measured mixture saturation temperature that is greater than a chosen reference temperature, highlights a lower solubility, and indicates formation of a stable cocrystal. These three techniques together are often selected due to their accessibility in research labs while utilizing very different cocrystallization mechanisms/pathways.

In this study, we aim to review the experimental screening methods by applying them in a wide screening protocol for PZQ cocrystals that involved 30 coformers selected using a network-based link prediction algorithm.^64−67^ Seventeen new multicomponent cocrystals of PZQ were identified, with 12 structures resolved. The coformer prediction method using network science and single-crystal structure characterizations is discussed in detail by Devogelaer et al^67^ In the present article, we focus on the cocrystal preparation and identification results that were obtained using the three different experimental methods: LAG, SE, and STM. Using our screening results, we provide a thorough comparison of experimental methods with quantified parameters that are (a) the fraction of screenable coformers, (b) the coformer success rate, (c) the ability to give several cocrystals per successful coformer, and (d) the identification of new stable phases. By comparing the methods’ parameters and their experimental convenience, we aim to conclude on their efficiency and provide relevant advice on optimization of cocrystal screening method selection.

## 2 Cocrystal Screening Methods

### 2.1 Materials and Experimental Protocols

(RS)-PZQ was provided by Merck KGaA (Darmstadt, Germany). The coformers used for screening are listed in the Supporting Information, with their purities and chemical suppliers in Tables S1 and S2 and their molecular structures in Figure S1. For SE and LAG experiments, the following solvents with purities higher than reagent grade were used: methanol, ethanol, isopropanol, acetonitrile, acetone, and ethyl acetate. Recently purchased ethanol, acetonitrile, and ethyl acetate with purities higher than 99% were used for the STM method to minimize the introduction of impurities and water.

### 2.2 X-ray Powder Diffraction

X-ray powder diffraction (XRPD) was used to identify a new phase by comparison with reference patterns of pure coformers. For clarity, figures in this article contain only the XRPD reference of stable polymorphs from pure starting coformers. LAG and SE samples were placed as a thin film of powder on zero-background (557)-silicon wafers and measured with a Malvern Panalytical Empyrean diffractometer. The diffractograms were measured in Bragg–Brentano geometry (reflection mode) using monochromatic Cu Kα radiation from a sealed LFF tube and using a PIXcel3D 1 × 1 detector. A continuous scan was performed in the 5° < 2θ < 30° range with a step size of 0.013° and a scan speed of 0.11° s^–1^. STM samples were analyzed using a Bruker D8 Advance II diffractometer with Debye–Scherrer transmission from Cu Kα source radiation (1.541 Å) with an operating voltage of 40 kV, current of 50 mA, a Kα1 Johansson monochromator, and an 1 mm antidivergence slit. A scanning range of 2θ values between 4 and 35° was applied with a scan speed of 0.017°s^–1^.

### 2.3 Solvent Selection and Pure Component Solubility Determination

A selection of solvents able to dissolve PZQ and coformers was required to perform LAG, SE, and STM cocrystal screening experiments. As most coformers from the list are to some extent polar, the following protic and aprotic polar solvents were chosen: methanol, ethanol (EtOH), isopropanol, acetonitrile (MeCN), acetone, and ethyl acetate (AcOEt), all commonly used in industry. For LAG and SE, the most appropriate solvent from this list was always chosen, that is, solubilizing but not too much. Experimental details can be found in Supporting Information (Table S3). For STM, only EtOH, MeCN, and AcOEt are selected as they present different chemical functions and can cope with experiments at temperatures higher than 60 °C. Saturation temperatures *T*_sat_ (i.e. solubility) of suspensions stirred at 700 rpm in 2 mL vials were measured using the Crystal16 (Technobis, Alkmaar, the Netherlands) system. The following temperature profile was used: dissolution at 60 °C followed by three cycles of cooling to −5 °C (−0.5 °C/min) and heating to 60 °C (0.3 °C/min), with isothermal periods of 90 min at −5 °C and 30 min at 60 °C. The clear point temperature in each cycle was identified as the temperature at which the light transmission passing through a sample reached 100%. The average of the three clear point temperatures was taken as the saturation temperature *T*_sat_ of the sample. The saturation temperatures were fitted with the Van‘t Hoff equation, allowing the estimation of any solubility of a pure component in the observed temperature range by using heat of fusion and melting temperature as fitting parameters. Solubility data and Van’t Hoff plots for PZQ and coformers can be found in Supporting Information (Section S3).

### 2.4 Cocrystal Preparation Methods

#### 2.4.1 LAG

Compositions screened with LAG contain amounts of solvent substantially lower than needed to dissolve the solid phases and undergo solid conversion without going through a clear solution state. About 50 mg of stoichiometric powders (1:1 molar ratio) containing PZQ and the coformer were ground in the presence of 40 μL of solvent in a Retsch MM 400 ball mill (Retsch GmbH, Haan, Germany). Grinding was performed in 1.5 mL stainless steel jars with one 5 mm stainless steel ball per jar for 30 min with a milling frequency of 25 Hz. Final solids were analyzed with XRPD.

#### 2.4.2 Solvent Evaporation

About 50 mg of a 1:1 stoichiometric mixture was prepared and dissolved in a solvent. The samples were then transferred to 10 mL glass vials, covered with parafilm in which five small holes were pierced with a needle, and left for complete evaporation of the solvent. The resulting solids were identified by XRPD.

#### 2.4.3 STM of Mixtures

While the LAG and SE methods use samples having an arbitrary stoichiometry in coformers to screen (1:1 in this study), the STM method uses stoichiometries determined by the pure component solubilities as the compositional range of the cocrystal stability domain in a solvent depends on these solubilities.^60^ When components have different solubilities, a stoichiometric solution for cocrystal preparation is indeed not optimal and can lead to missing a new cocrystal discovery.^53^ First, solubility curves of pure coformer and PZQ in the selected solvent were determined using the experimental protocol in Section 2.3. Then, reference temperatures *T*_r_ were chosen as working temperatures higher than room temperature to ensure the isolation of a solid phase at the end of the experiment. The pure PZQ and pure coformer solubility values at a reference temperature *T*_r_ were computed from the Van‘t Hoff plots obtained from solubility data. The computed concentrations in components were prepared experimentally to obtain samples with a stoichiometry described by the molar ratio *M*_PZQ/cof_ between the coformer and PZQ in solution. Finally, the screening was performed for each sample by measuring the experimental saturation temperature *T*_sat_ of the mixtures with the experimental protocol in Section 2.3. If *T*_sat_ measured for the mixtures are equal to the reference temperature *T*_r_ (ideal solution behavior) or lower (components influencing each other solubilities), it indicates that no new phase was formed. On the other hand, a saturation temperature *T*_sat_ higher than *T*_r_ for a mixture indicates the formation of a more stable cocrystal phase.^68^ The STM method gives therefore a quantified measurement on the existence of a new cocrystal phase. After the three temperature cycles, a final cooling to −5 °C (−0.5 °C/min) was performed and the crystallized material was collected for XRPD analysis to confirm the results.

## 3 Results

**Figure 2:**
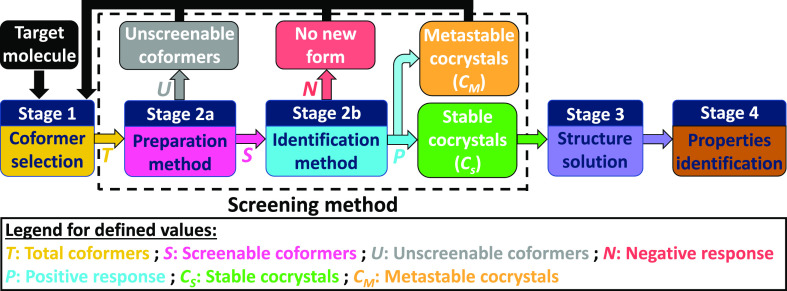
Schematic for cocrystal screening campaign stages, the strategy being to improve the properties of the target molecule by finding new stable cocrystals. In this article, we aim to compare different cocrystal screening methods (i.e., combination of preparation and identification techniques) by defining values resulting from stage 2. The values help to compute comparison parameters that we define as the screenable coformer fraction *R*_1_ (eq 1), the coformer success rate *R*_2_ (eq 2), the coformer pluriformity *R*_3_ (eq 3), and the new stable cocrystal coverage *R*_4_ (eq 4). We also compare methods based on their experimental convenience (time, cost, and equipment required).

The strategy of a cocrystal screening campaign is to improve the properties of a target molecule by finding new stable cocrystals (see Figure 2). In the case of PZQ, the aim is to find a cocrystal system permitting chiral separation. The first stage of cocrystal screening consists of selecting appropriate coformers likely to form cocrystals with the target molecule (Figure 2, stage 1). This work has been covered for this PZQ cocrystal screening campaign in an article from Devogelaer et al^67^ With a network-based link prediction algorithm for coformer selection using data mining techniques applied to the CSD, a list of 30 coformers was predicted and screened experimentally. The list of molecular structures and attributed ranks for each coformer can be found in Supporting Information (Section S1). The present study focuses on doing a thorough comparison of the results from screening methods LAG, SE, and STM to review their advantages and drawbacks (Figure 2, stage 2). The solved crystal structures of the newly found cocrystals through the screening campaign (Figure 2, stage 3) from single-crystal XRD information are detailed in the article from Devogelaer et al^67^

In this article, we define a cocrystal screening method as the combined process of attempting to produce a solid phase with a cocrystallization preparation method and determining its nature with an identification method that will measure if the produced solid mixture possesses new properties (Figure 2, stage 2). Preparation and identification can either be separated in the screening procedure or included in the same experiment depending on the screening method used. In the study, we identify LAG and SE as preparation methods only (stage 2a), and the prepared solids are measured by XRPD as an identification method (stage 2b) to assess cocrystal formation or not. However, the STM method directly results in an indication whether a new phase exists or not, since a mixture forming a stable cocrystal would result in a higher saturation temperature than expected for the pure single components.^60^ This means that STM is both a cocrystal preparation and an identification technique in the same experiment. Nonetheless, the new solid phases were also confirmed with XRPD for STM results.

To quantify and compare the effectiveness of the cocrystal screening methods, we propose to define quantified parameters calculated from the experimental data obtained through the different steps of Figure 2. Preparation methods can lead to some coformers not forming a suitable solid phase with the target molecule for later identification (amorphous or liquid) or to incompatibility with a method’s limitations, for instance, when showing thermal, chemical, or mechanical degradation or solvent incompatibility. Therefore, for each preparation method (LAG, SE, and STM) tried on the total number of coformers selected for screening (*T*), a certain number of coformers is considered screenable (*S*), while the rest is unscreenable (*U*) by that specific method. We define for each screening method its screenable coformer fraction parameter *R*_1_ (eq 1), that is, the fraction of coformers for which a solid phase could successfully be produced and analyzed with an identification technique.
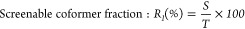
1

The prepared solids with screenable coformers are analyzed with an identification technique to determine if the measured properties are different from pure coformers or not. For these coformers, a part has a positive response to cocrystallization if at least one cocrystal is identified (*P*), and the other part has a negative response as no cocrystal is detected (*N*). We can then define a coformer success rate parameter *R*_2_ (eq 2) for each screening method.
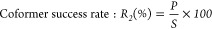
2

Newly identified cocrystals with one method can be stable when lower in energy than pure coformer mixtures, and we define their final number to be *C*_*S*_. Otherwise, they are metastable if at equilibrium they cannot be isolated due to acquisition of pure coformers instead, and we define this value to be *C*_*M*_ for that method. In this study, generally, single-crystal growth experiments and the different screening methods under varying conditions consistently led to the same form. In those cases, it is likely that the obtained form and thus also the obtained crystal structure^67^ is the stable form under the conditions of the experiment. However, these results do not guarantee that the new form is the thermodynamic stable form, and accurate stability studies in future research will have to confirm the hypotheses. One successful coformer can result in more than one new cocrystal identified, for instance, two cocrystals of different stoichiometries, different stabilities, or solvated or not. Therefore, we define a coformer pluriformity parameter *R*_3_ (eq 3) that quantifies a screening method’s ability to give more than one new cocrystal per successful coformer.
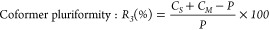
3

In the end of a cocrystal screening campaign, only new stable cocrystals found with one method (*C*_*S*_) are interesting in most cases for further research. By defining *C*^tot^ as the total of cocrystals (stable and metastable), found with all methods combined during the screening campaign, we characterize the new stable cocrystal coverage parameter *R*_4_ (eq 4) that describes the fraction of new stable cocrystals identified with one method.
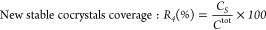
4

To review and compare the screening method results in the case of the present PZQ screening study, we use these defined parameters and discuss the methods’ convenience, in terms of experiment time, material cost, and equipment required.

### 3.1 LAG

The solvents used in LAG are listed in Supporting Information Table S3 and were chosen as being able to solubilize both PZQ and the coformer screened. LAG experiments typically result in a powder or a slurry that can then be analyzed with XRPD to identify potential cocrystal formation. However, for the three coformers 3-hydroxybenzoic acid (**16**), 3-nitrobenzoic acid (**25**), and 4-nitrophenol (**26**), LAG resulted in the formation of an oil or amorphous phase and the absence of a measurable XRPD pattern. These mixtures do not show crystallization even after a period of 90 days. Although these coformers have relatively high melting temperatures, an explanation could be that the binary melting temperatures of these coformers’ system are below the room temperature, preventing crystal formation as the melt would be the stable phase. Otherwise, crystallization kinetics of any solid phase could be very slow, resulting in an out-of-equilibrium phase. The cocrystal preparation experiments of these three coformer systems are inconclusive about cocrystal existence as no solid could be obtained for the XRPD analysis and hence are considered unscreenable with LAG. Therefore, *S* = 27 for the LAG method, setting its screenable coformer fraction parameter *R*_1_to be 90%.

**Figure 3:**
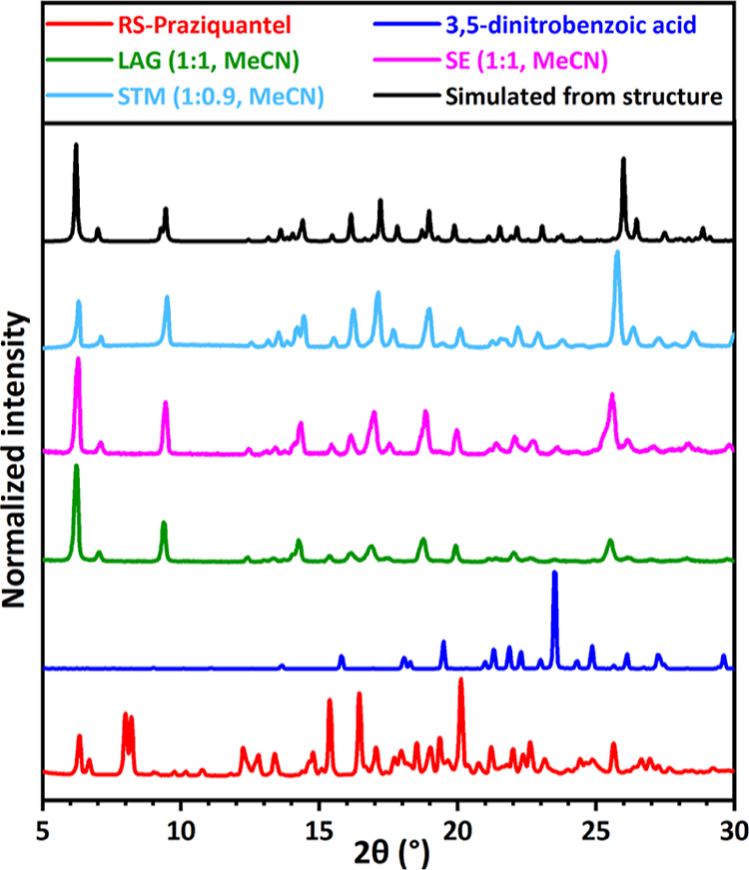
XRPD patterns for RS-PZQ, 3,5-dinitrobenzoic acid, and solid phases obtained from their mixtures after LAG, SE, and STM (with the corresponding solvent and molar ratio between the coformer and PZQ *M*_PZQ/cof_). The simulated powder pattern from the resolved cocrystal (CCDC 2054491^67^) is added for comparison. This new pattern is identified for the LAG, SE, and STM experiments.

**Figure 4:**
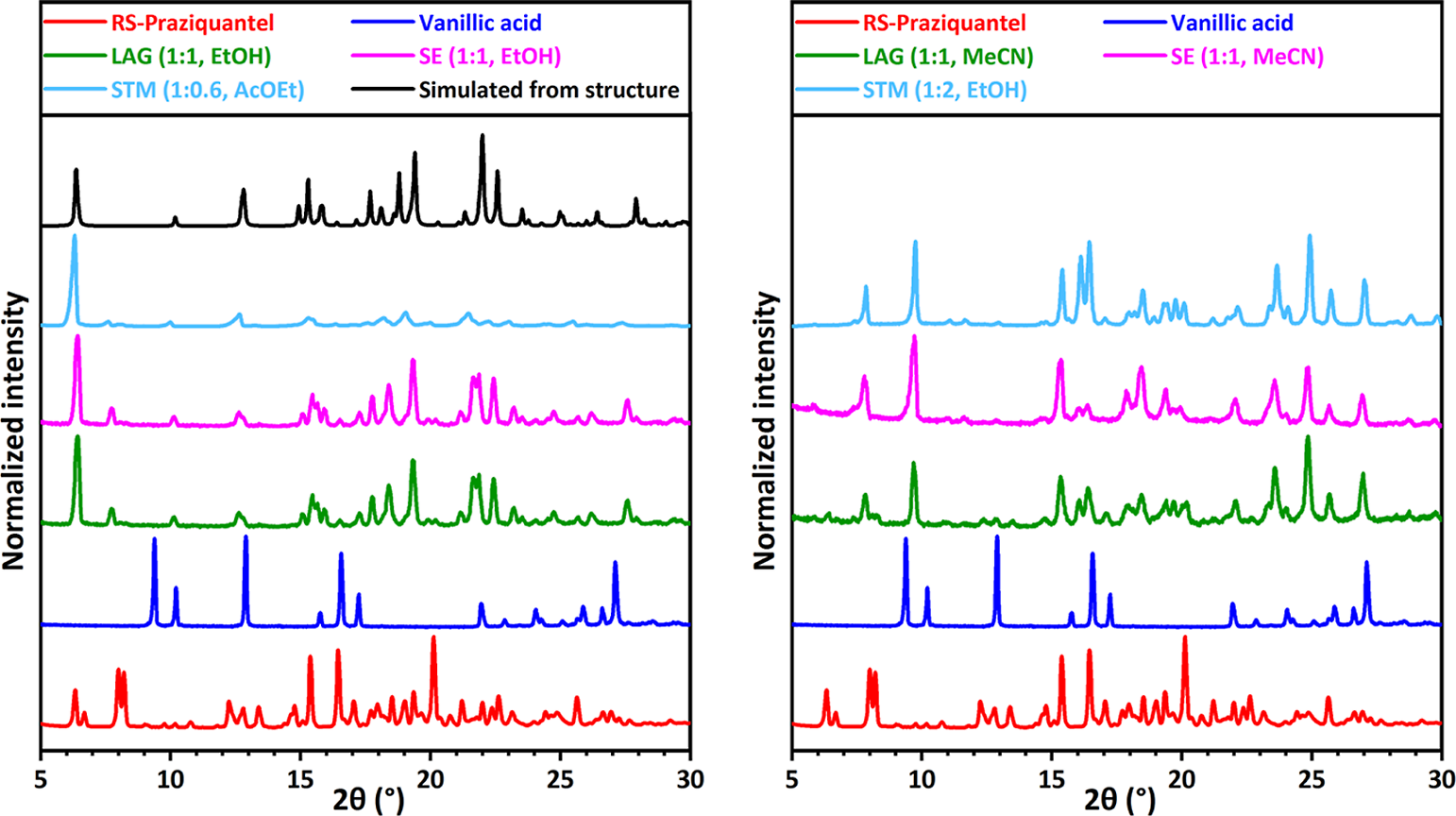
XRPD patterns for RS-PZQ, vanillic acid (**20**), and solid phases obtained from their mixtures after LAG, SE, and STM (with the corresponding solvent and molar ratio M_PZQ/cof_ between the coformer and PZQ). The simulated powder pattern from the resolved cocrystal (CCDC 2054490^67^) is added for comparison. Two new patterns, one presented in the left graph and the other in the right graph, are identified for LAG, SE, and STM depending on the solvent used.

**Figure 5:**
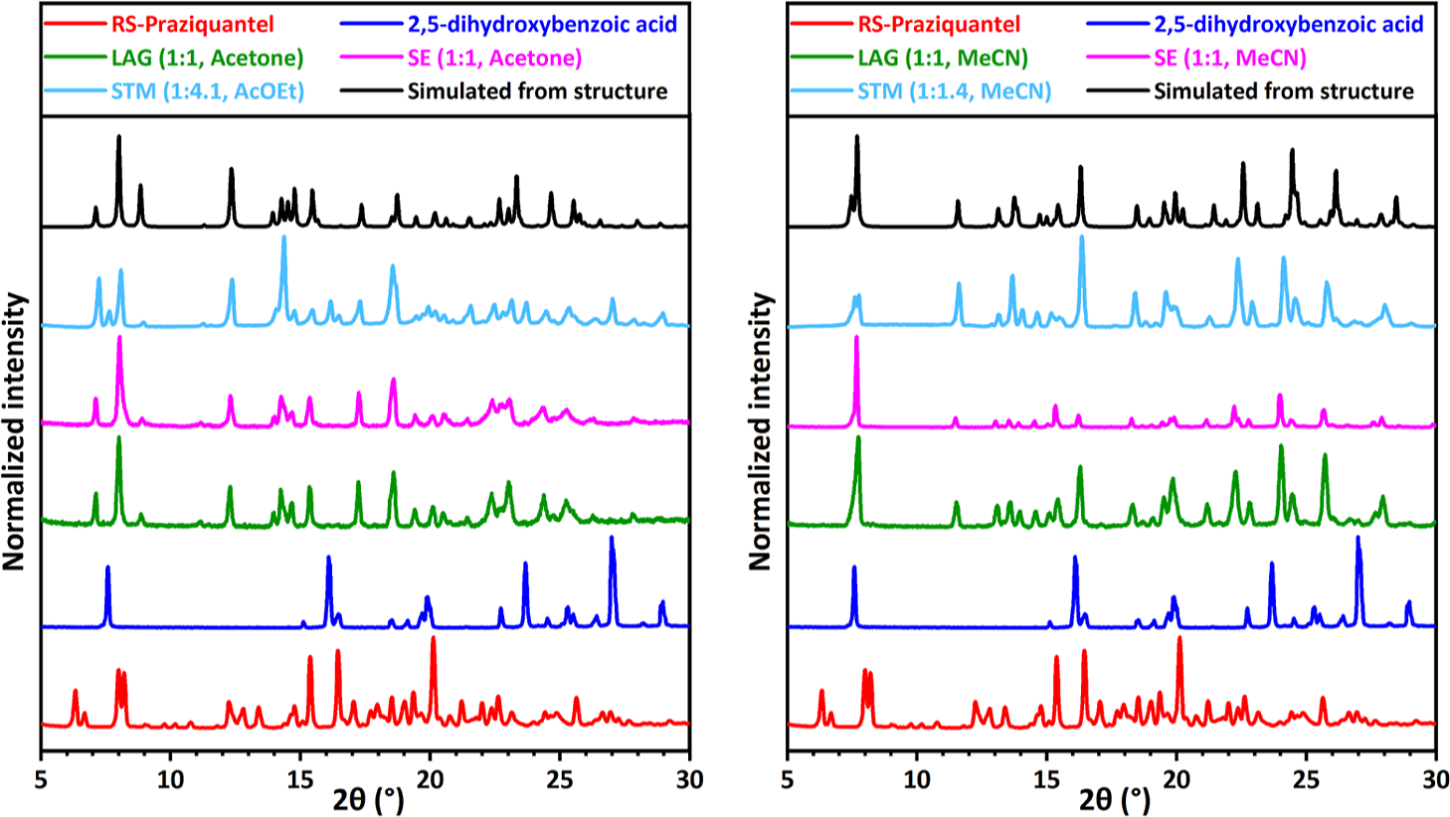
XRPD patterns for RS-PZQ, 2,5-dihydroxybenzoic acid, and solid phases obtained from their mixtures after LAG, SE, and STM (with the corresponding solvent and molar ratio M_PZQ/cof_ between the coformer and PZQ). The simulated powder patterns from the resolved cocrystal (CCDC 2054489,^67^ left) and the resolved cocrystal solvate (CCDC 2054487,^67^ right) are added for comparison. Two new patterns, one presented in the left graph and the other in the right graph, are identified for LAG, SE, and STM depending on the solvent used.

**Figure 6:**
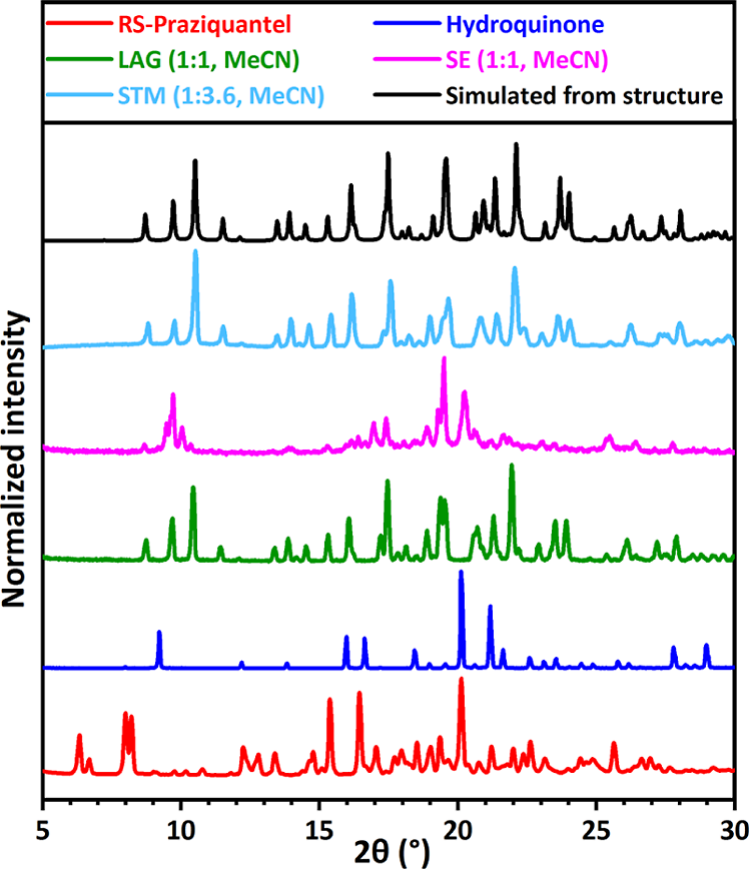
XRPD patterns for RS-PZQ, hydroquinone, and solid phases obtained from their mixtures after LAG, SE, and STM (with the corresponding solvent and molar ratio M_PZQ/cof_ between coformer and PZQ). The simulated powder pattern from the resolved cocrystal (CCDC 2054497^67^) is added for comparison. This new pattern is identified for LAG and STM. SE presents a different new pattern (corresponding structure has not been characterized; so no simulated powder pattern is shown).

With LAG experiments, 11 coformers out of the 27 screenable ones show a positive response in cocrystallization (*P* = 11), setting the coformer success rate parameter *R*_2_to be 41%. As an example of positive screening experiment, the coformer 3,5-dinitrobenzoic acid (**13**), shown in Figure 3 (green), indicates a significantly different XRPD pattern compared to that of the pure coformer (dark blue) and PZQ (red). We note a complete conversion into the new phase as there is no trace of either the pure coformer or PZQ peaks in the pattern. New patterns are also identified for salicylic acid (**5**, Figure S3), 1,4-diiodotetrafluorobenzene (**6**, Figure S4), 4-hydroxybenzoic acid (**7**, Figure S5), 4-aminosalicylic acid (**12**, Figure S6), hydroquinone (**15**, Figure 6 green), vanillic acid (**20**, Figure 4 green), 2,5-dihydroxybenzoic acid (**22**, Figure 5 green), 3,5-dihydroxybenzoic acid (**24**, Figure S13), 2,4-dihydroxybenzoic acid (**28**, Figure 9 green), and orcinol (**29**, Figure S16). No evidence of cocrystal formation is found for 16 other screened coformers as the XRPD patterns indicate the presence of already known solid phases from coformers and PZQ (*N* = 16).

In most cases, systems screened with LAG in multiple solvents result in the same solid phase formation. However, XRPD patterns can differ depending on the solvent used. This is the case in our study of PZQ and vanillic acid (**20**) which gives different XRPD patterns in LAG for EtOH and MeCN, as shown in green patterns in Figure 4. Another example is 2,5-dihydroxybenzoic acid (**22**), as shown in Figure 5, that has two new, different patterns with LAG in acetone and MeCN (green). Possible explanations are the formation of a cocrystal and a cocrystal solvate or two stable cocrystals of different stoichiometries or a stable cocrystal and a metastable cocrystal of the same stoichiometry (polymorphism). In total, 13 new cocrystal XRPD patterns are identified using LAG for 11 positive coformers, setting its coformer pluriformity parameter *R*_3_to be 18%.

The solved crystal structures from single-crystal X-ray diffraction information help to conclude on the nature of the new crystals formed, that is,: cocrystal, cocrystal solvate, and their stoichiometries. Among the 13 new XRPD patterns identified using LAG, single-crystal growth experiments confirm 12 new cocrystal structures where the simulated patterns correspond to those obtained from the LAG experiments. Eight coformers are identified as forming 1:1 molar stoichiometry cocrystals with PZQ: 1,4-diiodotetrafluorobenzene (**6**), 4-hydroxybenzoic acid (**7**), 4-aminosalicylic acid (**12**), hydroquinone (**15**), vanillic acid (**20**), 2,5-dihydroxybenzoic acid (**22**), 2,4-dihydroxybenzoic acid (**28**), and orcinol (**29**). Four coformers are identified as forming 1:1:1 cocrystal solvates with PZQ and a solvent. Three of these solvates are with MeCN: 4-aminosalicylic acid (**12**), 2,5-dihydroxybenzoic acid (**22**), and 3,5-dihydroxybenzoic acid (**24**). The fourth is a cocrystal hydrate unexpectedly obtained with salicylic acid (**5**) even if water is not used here as a solvent. Indeed, acetone is used in this case for grinding, which leads to an oil transition stage that crystallizes upon contact with ambient humidity from air. Two distinctly new XRPD patterns, presented in Figure 4, were obtained using the coformer vanillic acid in LAG. The phase produced in LAG using the solvent EtOH (green, left) is a 1:1 cocrystal, whose structure is solved by single-crystal XRD. The other phase was produced in LAG using the solvent MeCN (green, right), but the single-crystal growth experiments were not successful in producing this cocrystal form.

### 3.2 SE

SE experiments require solvents in which both the coformer and PZQ have a substantial solubility and evaporate relatively quickly under ambient conditions. The solvents were screened, and those used for SE for each coformer are listed in Supporting Information Table S3. Three coformers, namely, terephthalic acid (**8**), isophthalic acid (**10**), and phthalic acid (**18**), do not have a suitable solvent as only DMF is found to dissolve them but does not evaporate under ambient conditions. Therefore, these coformers are unscreenable by the SE method due to solvent incompatibility. The other coformers were tested for solid mixture preparation. Successful preparation attempts result in a powder or a slurry that can be analyzed with XRPD to confirm cocrystal formation. Nine coformer systems, namely, benzoic acid (**3**), *trans*-cinnamic acid (**14**), 3-hydroxybenzoic acid (**16**), anthranilic acid (**17**), d-tartaric acid (**19**), 3-nitrobenzoic acid (**25**), 4-nitrophenol (**26**), 1-hydroxy-2-naphtoic acid (**27**), and orcinol (**29**), result in oils/amorphous materials and therefore no solid phases identifiable with XRPD analysis. It is unlikely that after complete evaporation, the stable equilibrium for these mixtures is the liquid state at room temperature. Therefore, these issues are due to fast crystallization kinetics caused by fast evaporation resulting in an amorphous state or due to trapping of the remaining solvent in a liquor that becomes too viscous to permit complete evaporation. These coformers are also considered unscreenable as SE preparation attempts are unsuccessful, and no conclusion about cocrystal existence for these systems is possible. Therefore, *S* = 18 for the SE method, setting its screenable coformer fraction parameter *R*_1_to be 60%.

With SE experiments, 10 coformers out of the 18 screenable ones show a positive response in cocrystallization (*P* = 10), setting the coformer success rate parameter *R*_2_to be 56%. It includes the result of the coformer 3,5-dinitrobenzoic acid (**13**) with PZQ, as shown in Figure 3 (pink). This new pattern is the same as the one obtained with LAG for this system (green). New patterns are also identified for PZQ with pimelic acid (**4**, Figure S2), salicylic acid (**5**, Figure S3), 1,4-diiodotetrafluorobenzene (**6**, Figure S4), 4-hydroxybenzoic acid (**7**, Figure S5), hydroquinone (**15**, Figure 6 pink), vanillic acid (**20**, Figure 4 pink), 2,5-dihydroxybenzoic acid (**22**, Figure 5 pink), 3,5-dihydroxybenzoic acid (**24**, Figure S13), and 2,4-dihydroxybenzoic acid (**28**, Figure 9 pink). No evidence of cocrystal formation is found for the eight other screened coformers as the XRPD patterns indicate the presence of already known solid phases from coformers and PZQ (*N* = 8).

In total, 12 new cocrystal XRPD patterns are identified using SE for 10 positive coformers, setting its coformer pluriformity parameter *R*_3_to be 20%. As in the case of LAG, the two coformers, vanillic acid (**20**, Figure 4) and 2,5-dihydroxybenzoic acid (**22**, Figure 5) give new XRPD patterns that depend on the solvent used. The same solvents with the same 1:1 ratio between PZQ and the coformer are used in LAG and SE, so the results are consistent between the two methods.

**Figure 7:**
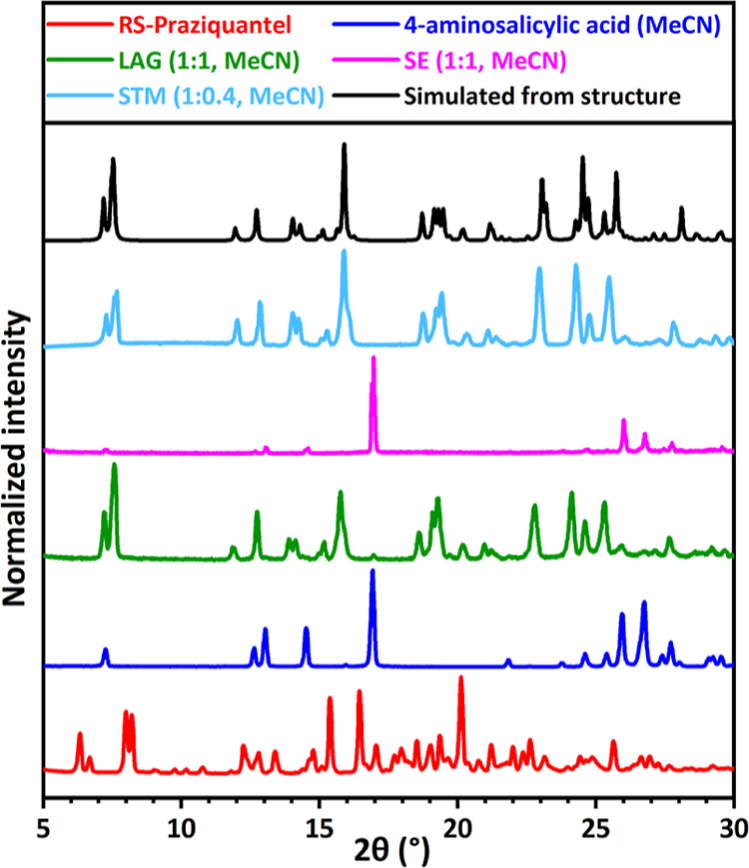
XRPD patterns for RS-PZQ, 4-aminosalicylic acid, and solid phases obtained from their mixtures after LAG, SE, and STM (with the corresponding solvent and molar ratio *M*_PZQ/cof_ between the coformer and PZQ). The simulated powder pattern from the resolved cocrystal solvate (CCDC 2054493^67^) is added for comparison. This new pattern is identified for LAG and STM but not SE.

However, the new XRPD patterns with SE for hydroquinone (**15**, Figure 6 pink) and 2,4-dihydroxybenzoic acid (**28**, Figure 9 pink) are not the same as the 1:1 cocrystals obtained with LAG (green), whose stabilities are indicated from consistent results with single-crystal growth experiments. These different patterns from SE results are observed, despite SE experiments being done in the same solvent with the same equimolar ratio as with LAG. No single crystals could be grown for these phases as growth experiments result in the LAG cocrystals suspected to be stable and not the SE phases. The same problem is encountered for pimelic acid using SE (**4**, Figure S2), with a new pattern identified that shows the pimelic acid pattern containing an additional peak not corresponding to any known phase. No new pattern is identified with LAG under the same experimental conditions, and growth experiments lead to pure coformer phases and not the solid identified with SE. Therefore, the question about the nature of these three phases remains, and as they are different from the known pure coformer solids, our interpretation is that they are metastable cocrystals/cocrystal solvates. For the other coformers having a positive response to cocrystallization, the XRPD patterns with SE correspond to the same as those identified with LAG from which indications of stability are obtained from single-crystal growth experiments. However, for 4-aminosalicylic acid (**12**) in Figure 7 the SE result (pink) indicates no cocrystal formation as pure 4-aminosalicylic acid is obtained (dark blue pattern), contrary to LAG for the same composition.

### 3.3 STM

For STM experiments, it is necessary to find a solvent that solubilizes both the coformer and PZQ. Pure component solubility curves are acquired to choose the optimal mixture composition screened. This composition corresponds to the pure component solubilities at a reference temperature *T*_r_, chosen arbitrarily at a temperature higher than room temperature to ensure obtaining a solid phase. The screening is done in more than one solvent, up to a maximum of three solvents, which leads to mixture molar ratios *M*_PZQ/cof_ that vary with the solvent used. The screening strategy for STM consists of first measuring coformer solubility curves for which the Van’t Hoff plots are presented in Figures S17–S42 with related data Tables S4–S29 in Supporting Information Section S3. Then, mixtures with PZQ and the coformer are screened using the following order of solvents: EtOH, MeCN, and AcOEt. Five coformer systems could not be screened with these solvents, namely, terephthalic acid (**8**), isophthalic acid (**10**), phthalic acid (**18**), d-tartaric acid (**19**), and orcinol (**29**), due to solubility issues. All solvents tried could not dissolve **8**, **10**, and **18**. Solutions of **29** did not crystallize. Only EtOH could dissolve **19**, but the solubility measurements resulted in inconsistent despite multiple experiments. These coformer systems, for which no pure component solubility data can be obtained, are considered unscreenable with STM because of solvent incompatibility. Therefore, *S* = 25 for the STM method, setting its screenable coformer fraction parameter *R*_1_to be 83%.

**Figure 8:**
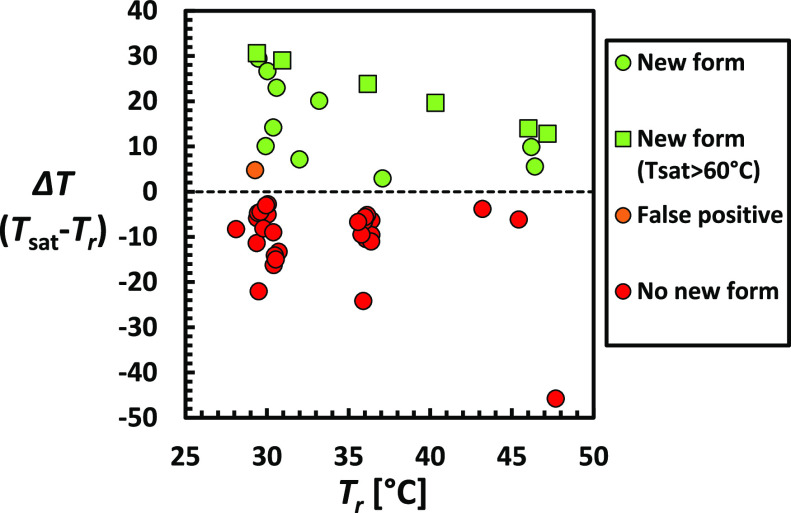
Temperature difference Δ*T* = *T*_sat_ – *T*_r_ versus the reference temperature T_r_ for systems investigated with the STM method. *T*_sat_ is the saturation temperature of mixtures containing PZQ and the coformer, both with a concentration equal to their ideal solubility at the chosen reference temperature *T*_r_ in studied solvents. A positive value of Δ*T* indicates potential formation of stable cocrystals, which is confirmed by XRPD (green). The STM method applied to 30 coformers results in one false positive (orange) when a positive Δ*T* is obtained, but XRPD indicates a coformer physical mixture. No false negatives, that is, cocrystals confirmed by XRPD with Δ*T* < 0, are observed. Crystallization of coformer physical mixtures (red) correspond to Δ*T* values below 0.

**Figure 9:**
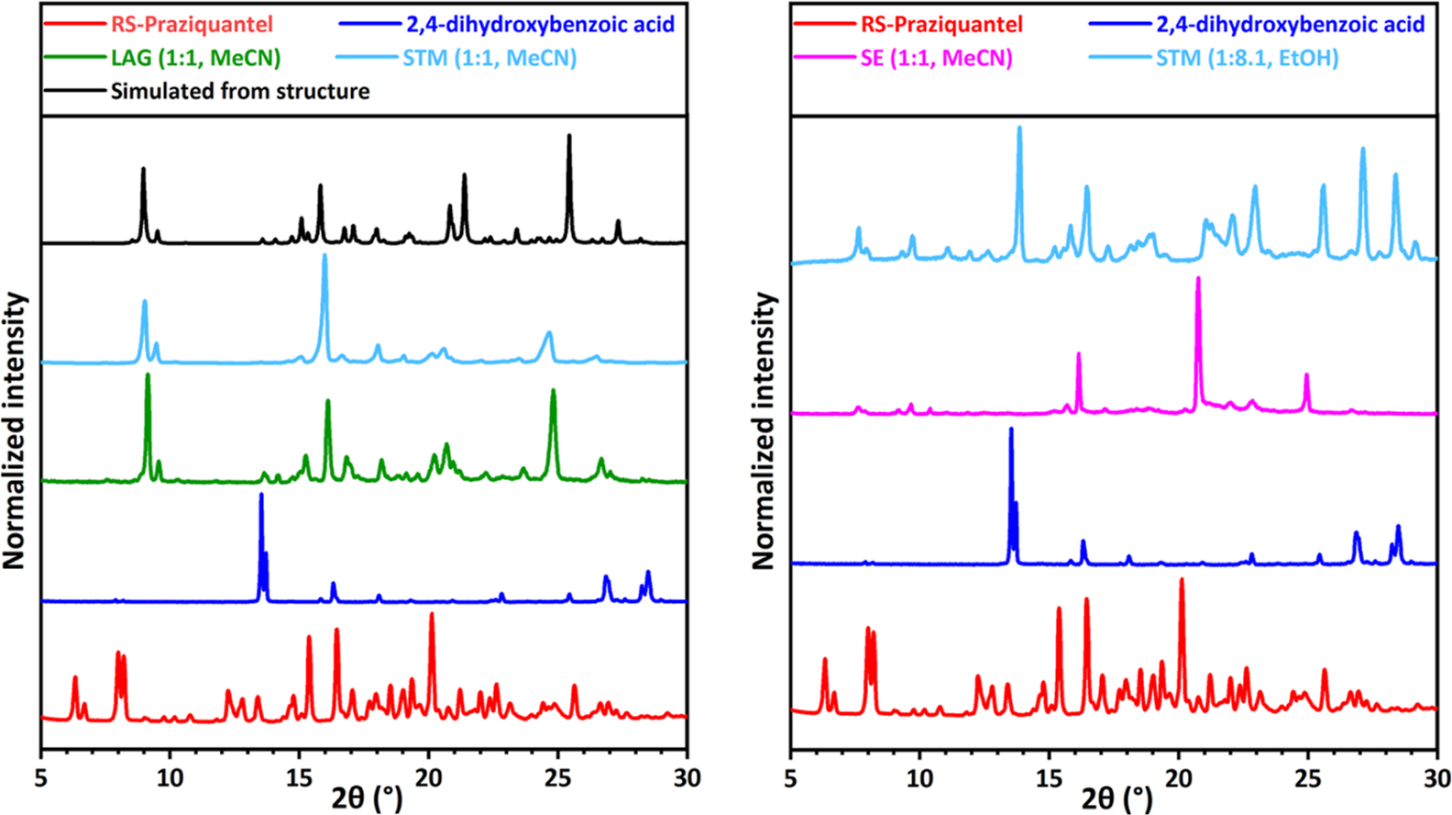
XRPD patterns for RS-PZQ, 2,4-dihydroxybenzoic acid, and solid phases obtained from their mixtures after LAG, SE, and STM (with the corresponding solvent and molar ratio *M*_PZQ/cof_ between the coformer and PZQ). The simulated powder pattern from the resolved cocrystal (CCDC 2054494^67^) is added for comparison. This new pattern is identified for LAG and STM (left). New different patterns are also identified for SE and STM in other conditions (right), which differ from it. SE phase is suspected to be metastable.

Screening experiment details are given in Supporting Information (Table S30) that summarizes screened compositions by the STM method with the corresponding molar ratio *M*_PZQ/cof_ between the coformer and PZQ in solution. The results are indicated by the temperature difference Δ*T* = *T*_sat_ – *T*_r_ between the measured saturation temperature *T*_sat_ of the mixture and the reference temperature *T*_r_. As represented in Figure 8, the newly identified cocrystals by the STM method show a positive Δ*T*, which is a strong thermodynamic indication of the formation of a more stable phase that is less soluble than both pure components. For example, a screened sample in MeCN with a concentration in PZQ of 0.3168 and 0.2834 mol/L in 3,5-dinitrobenzoic acid experimentally dissolves at a measured *T*_sat_ = 53.6 °C. From the Van‘t Hoff plots of pure component solubility data in MeCN, a solution of pure PZQ with a concentration of 0.3168 mol/L dissolves at 30.2 °C and a solution of pure 3,5-dinitrobenzoic acid with a concentration of 0.2834 mol/L dissolves at 30.6 °C. The reference temperature *T*_r_ is defined as the highest between both, and therefore *T*_r_ = 30.6 °C, giving a positive Δ*T* = 23 °C for this system that assesses the formation of a cocrystal less soluble than both pure components. XRPD analyses of the samples giving positive Δ*T* confirm the formation of cocrystals with new patterns and assess the method’s reliability (Figure 8, green data). When multiple experiments on the same coformer are performed in different solvents or molar ratio *M*_PZQ/cof_, XRPD also allows to know the new solid phase it concerns. This is not the case if only saturation temperatures are used as the latter indicate cocrystal formation but do not consist of a solid form fingerprint contrary to XRPD patterns. With STM experiments, 9 coformers out of the 25 screenable ones show a positive response in cocrystallization (*P* = 9), setting the coformer success rate parameter *R*_2_to be 36%. This involves 1,4-diiodotetrafluorobenzene (**6**, Figure S4), 4-hydroxybenzoic acid (**7**, Figure S5), 4-aminosalicylic acid (**12**, Figure 7 light blue), 3,5-dinitrobenzoic acid (**13**, Figure 3 light blue), hydroquinone (**15**, Figure 6 light blue), vanillic acid (**20**, Figure 4 light blue), 2,5-dihydroxybenzoic acid (**22**, Figure 5 light blue), 3,5-dihydroxybenzoic acid (**24**, Figure S13), and 2,4-dihydroxybenzoic acid (**28**, Figure 9 light blue). No evidence of cocrystal formation is found for the 16 other screened coformers as the XRPD patterns indicate the presence of already known solid phases from coformers and PZQ (*N* = 16).

A false positive is observed for benzoic acid (**3**) in EtOH with a positive temperature difference of Δ*T* = 4.7 °C, while XRPD confirms a physical mixture of PZQ and benzoic acid (Figure 8, orange circle). This is probably caused by a decrease in the solubility of one component due to the other. Systems for which the measurement of Δ*T* is below 0 show negative response to cocrystallization (Figure 8, red data) and correspond to components enhancing each other’s solubilities with favorable interactions. Sometimes, the effect can be substantial, for instance with 2,5-dihydroxybenzoic acid (**22**) in MeCN for which a temperature difference of Δ*T* = −45.8 °C is measured. XRPD of the solids corresponding to red data always consists of pure coformers. In some cases, crystallization did not happen, and no *T*_sat_ data or solid phases for XRPD could be obtained: **5** in MeCN, **9** in AcOEt, **16** in EtOH/MeCN/AcOEt, **17** in EtOH/MeCN, **21** in AcOEt, and **27** in AcOEt. These experiments are considered to not result in cocrystallization and to correspond to more extreme cases of overall enhanced solubility when mixing components.

The Δ*T* for newly identified form systems varies based on the relative stabilities of the new phases, going from Δ*T* = 7.1 °C for a cocrystal solvate with MeCN and 4-aminosalicylic acid (**12**) to Δ*T* = 29.4 °C for a cocrystal with 2,4-dihydroxybenzoic acid (**28**). For some systems, the saturation temperature is so high (beyond the boiling point of the solvent) that it could not be measured, such as cocrystals with 1,4-diiiodotetrafluorobenzene (**6**) in EtOH, 4-hydroxybenzoic acid (**7**) in MeCN, 2,5-dihydroxybenzoic acid (**22**) in MeCN, and 2,4-dihydroxybenzoic acid (**28**) in MeCN and AcOEt. To nevertheless show these experiments in Figure 8 (square symbols), their *T*_sat_ is assumed to be 60 °C, the maximum temperature in the temperature profiles. This highlights the accuracy of the detection method as stable cocrystals will be less soluble than the coformer mixture. The STM is then sufficient proof of a stable cocrystal formation, as no false negatives, that is, cocrystals confirmed by XRPD but with Δ*T* < 0, are observed.

In total, 12 new cocrystals are identified using STM for 9 positive coformers, setting its coformer pluriformity parameter *R*_3_to be 33%. As in the case of LAG and SE, the two coformers, vanillic acid (**20**, Figure 4) and 2,5-dihydroxybenzoic acid (**22**, Figure 5), give new XRPD patterns that depend on the solvent used. This is the same for 2,4-dihydroxybenzoic acid (**28**, Figure 9 right, light blue), whose new pattern obtained in EtOH is specific to STM. As no single crystal was grown for this phase, it is unclear whether it is a cocrystal of a different stoichiometry than the one confirmed by LAG or a cocrystal solvate with EtOH. However, its solubility is much lower than the pure component mixture, indicated by a high Δ*T* = 29.4 °C, which is a good indication of its stable nature. For the other coformers indicating positive response to cocrystallization, the XRPD patterns with STM correspond to the same as those identified with LAG from which stable cocrystals are suspected. The pattern of PZQ with 2,5-dihydroxybenzoic acid in AcOEt prepared with the STM method (Figure 5, left) presents extra peaks corresponding to the pure coformer pattern compared to the cocrystal simulated pattern. As the solid sample was taken from the suspension at a temperature well below its saturation temperature, the mixed XRPD pattern indicates that the sample equilibrated in a triphasic stability domain and not as a pure cocrystal in suspension.

### 3.5 Overview of Screening Results

**Figure 10:**
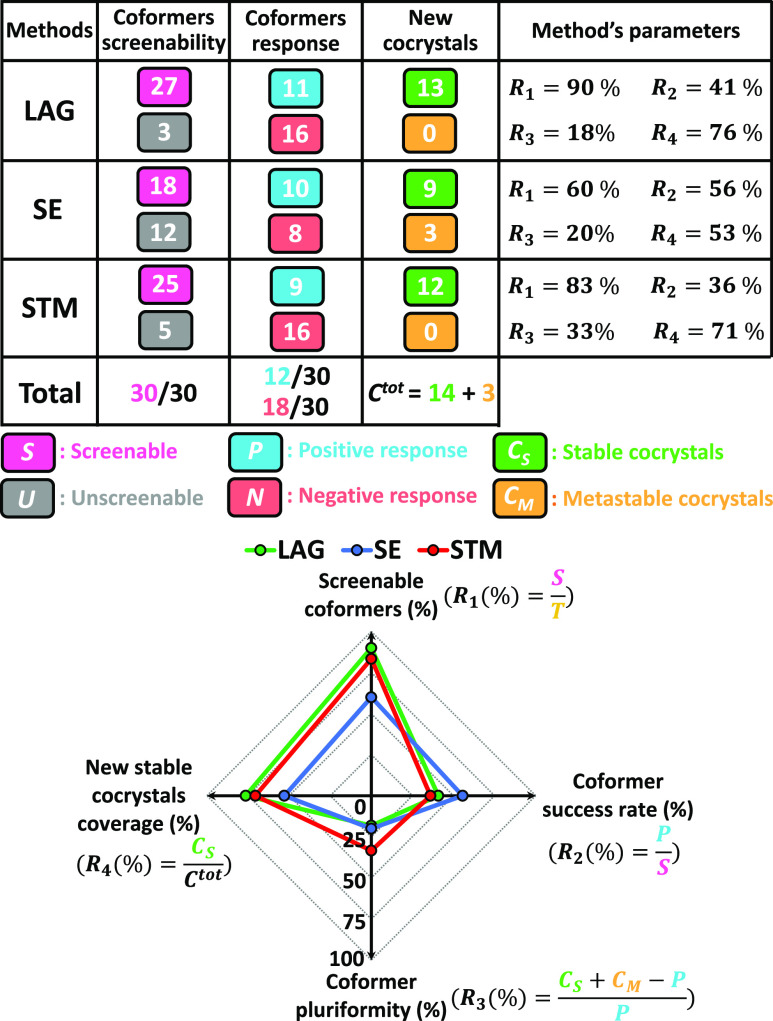
Overview of screening results per method with quantified parameters defined for comparison plotted as a web chart.

Of the 30 coformers selected, all could be screened, and 12 indicate a positive response to cocrystallization with PZQ for a total of 17 cocrystals found with all methods combined (*C*^tot^ = 17). Table 1 summarizes the screening results for each coformer, and Figure 10 represents an overview of all values obtained for the methods, with their computed parameters for comparison. The right column in Table 1 contains the information of the XRPD patterns. The same XRPD patterns are identified by all methods presenting a tick. If distinctly different XRPD patterns were obtained, the additional tick is explained in the right column of Table 1 to clarify by what method the phase was identified. We have indications from single-crystal growth experiments that 12 cocrystalline phases might be stable, of which the structures are reviewed in the article by Devogelaer et al^67^ All 12 cocrystals are identified with LAG, and the one found for orcinol (**29**, Figure S16) is specific to LAG. With LAG, a first cocrystal for vanillic acid (20, Figure 4 left) is identified, the structure of which is shown from single-crystal XRD information to correspond to a 1:1 cocrystal. However, a second cocrystal for vanillic acid (**20**, Figure 4 right) is also discovered, whose structure was not resolved. As this second result is obtained multiple times using LAG, SE, and STM in different solvents with varied compositions, we assume that the most likely hypothesis is a second stable cocrystal with a stoichiometry different from 1:1. The possibility of a cocrystal solvate is indeed excluded as the result in obtained in multiple solvents. Also, the consistent and repeated results obtained with all methods, and particularly STM that provides equilibrated suspensions, indicate the stable nature of this second cocrystal. The stoichiometry of this cocrystal could probably be two vanillic acid molecules for one PZQ as it is prepared with the STM method in solution composition in EtOH with an excess of vanillic acid compared to PZQ (Figure 4). Therefore, *C*_*S*_ = 13 for LAG, and this sets its new stable cocrystal coverage parameter *R*_4_ to be 76%. With SE, 12 new cocrystals are identified, with 9 in common with LAG from which we have stability indication due to consistent results with LAG, STM, and single-crystal growth experiments. The three others correspond to specific cocrystals identified with SE for pimelic acid (**4**, Figure S2), hydroquinone (**15**, Figure 6), and 2,4-dihydroxybenzoic acid (**28**, Figure 9). These phases are considered metastable due to inconsistency with LAG and STM experiments in similar conditions, as well as single-crystals growth experiments giving more stable phases instead. Therefore, *C*_*M*_ = 3 and *C*_*S*_ = 9 for SE, and it sets its new stable cocrystal coverage parameter *R*_4_ to be 53%. With STM, 12 new cocrystals are identified, with 11 in common with LAG from which we have stability indication due to consistent results with LAG, SE, and single-crystal growth experiments. A second cocrystal is obtained for 2,4-dihydroxybenzoic acid in EtOH (**28**, Figure 9), being a specific result to STM. Consistent experiments and a high Δ*T* value give indications of its stability, although screening experiments alone do not permit conclusions on its stoichiometry and eventual solvation. Therefore, *C*_*S*_ = 12 for STM, and this sets its new stable cocrystal coverage parameter *R*_4_ to be 71%.

**Table 1:**
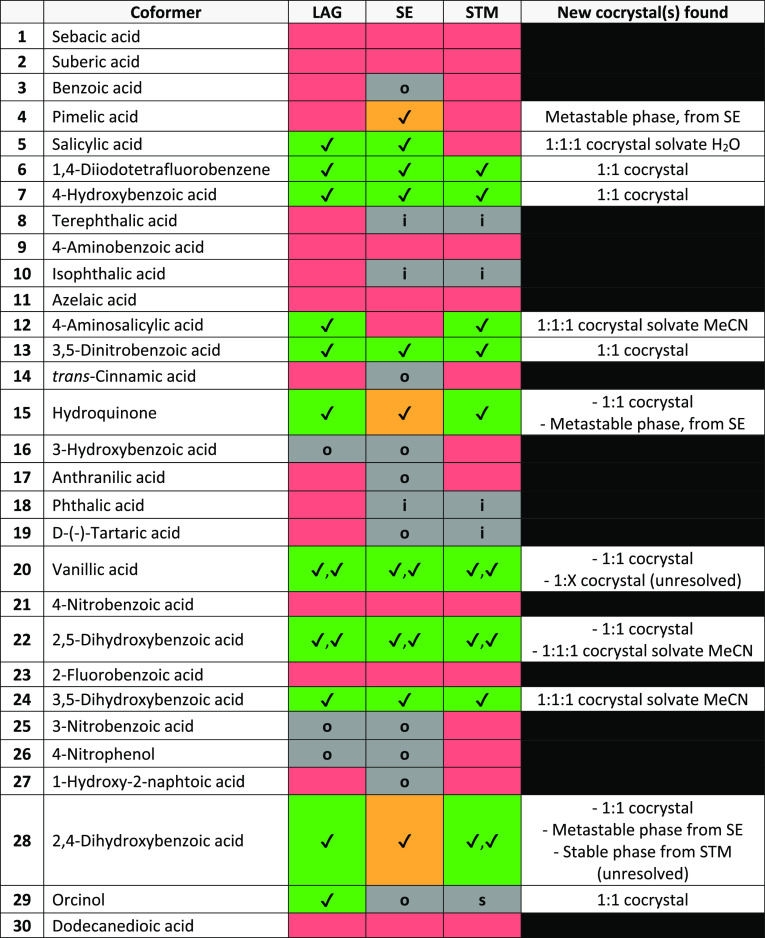
Coformer Screening Results With LAG, SE, and STM Methods^a^

a √: New XRPD pattern. Green: cocrystals showing stability. Red: physical mixture of coformers. Orange: cocrystals suspected to be metastable. Gray: unscreenable because insoluble (i), too soluble (s) or forming oils/amorphous (o).

In our study, we did not obtain a cocrystal during LAG of PZQ and pimelic acid in the presence of MeCN, contrary to the LAG experiments reported by Espinosa-Lara et al.,^40^ but rather a physical mixture of the raw materials (**4**, Figure S2). The XRPD pattern of our SE experiments, however, contains new diffraction peaks next to those of pimelic acid, although they are different from Espinosa-Lara et al.’s result. As we conclude on the metastability of the latter phase in our experiments, it remains unclear if a stable cocrystal with pimelic acid exists. Similarly, we did not obtain a cocrystal phase for d-tartaric acid with LAG reported by Cugovcan et al.^41^ We also do not observe the recent results from Liu et al.^69^ reporting cocrystals of PZQ with 3-hydroxybenzoic acid (**16**) and phthalic acid (**18**) prepared from dissolution in hot solvent followed by evaporation. Phthalic acid (**18**) is screened in our study with LAG and results in no new form, though it is uncertain if the cocrystal preparation method was efficient due to the insolubility of phthalic acid in the chosen solvents. 3-Hydroxybenzoic acid (**16**) is screened with STM using three different solvents in a total of six experiments with varying molar ratios *M*_PZQ/cof_ from 1:0.8 to 1:7.7, and all show the absence of crystallization in solution upon cooling, even at the low temperature.

## 4 Discussion

**Figure 11:**
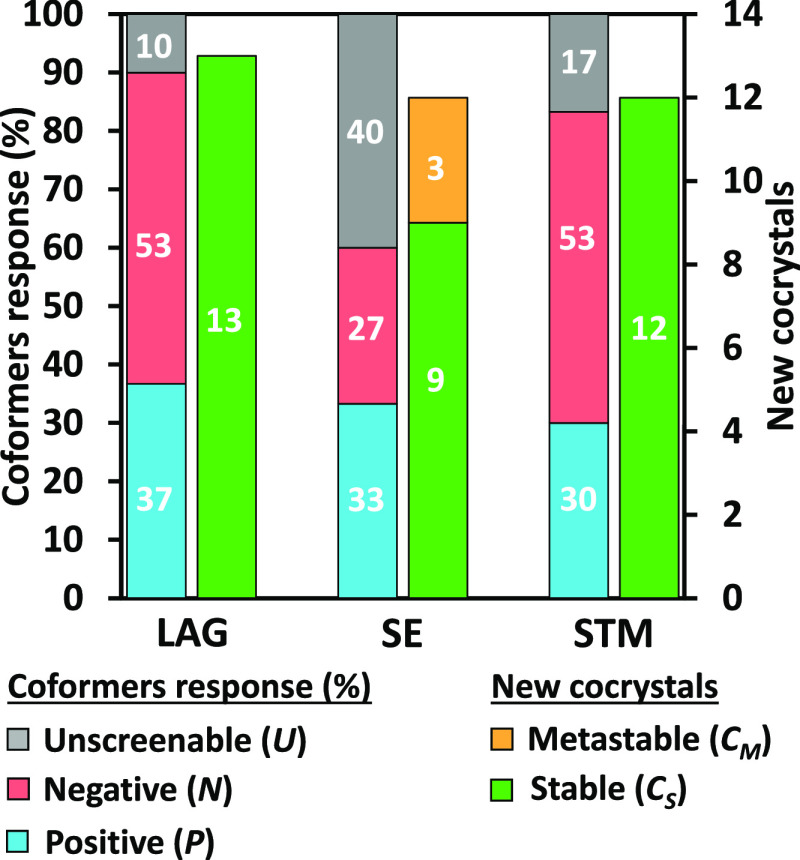
Stacked bar chart representing the ratio of positive (light blue), negative (red), and unscreenable (gray) responses for the screening methods used and their identified cocrystal number.

**Figure 12:**
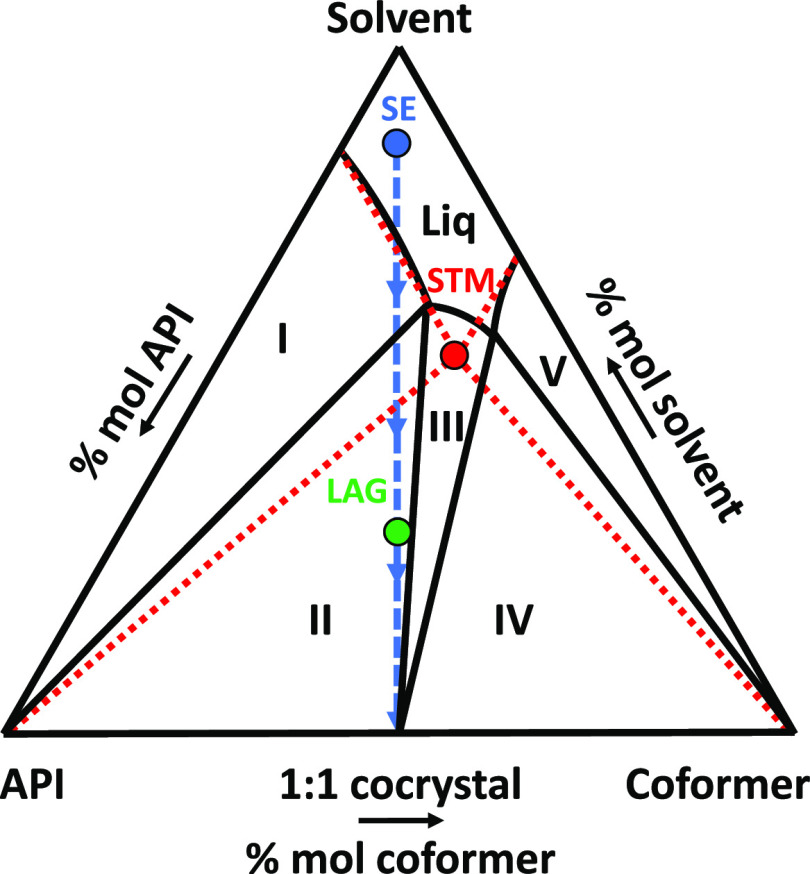
Schematic isothermal ternary phase diagram describing a 1:1 cocrystal forming system with noncongruent solubility. Regions I, III, and V are stability domains of the API, the cocrystal, and the coformer, respectively. Regions II and IV are triphasic domains between the cocrystal and a solution of eutectic composition and the API and the coformer, respectively. Liq stands for the undersaturated solution domain. The red point is the theoretical eutectic composition at that specific temperature (solution doubly saturated in API and coformer, computed from pure components ideal solubilities) chosen as the screening composition for the STM method. The green point corresponds to an arbitrary stoichiometric ratio screened by the LAG method. The blue dashed line corresponds to the crystallization pathway with the SE method from a stoichiometric undersaturated solution (blue point).

**Figure 13:**
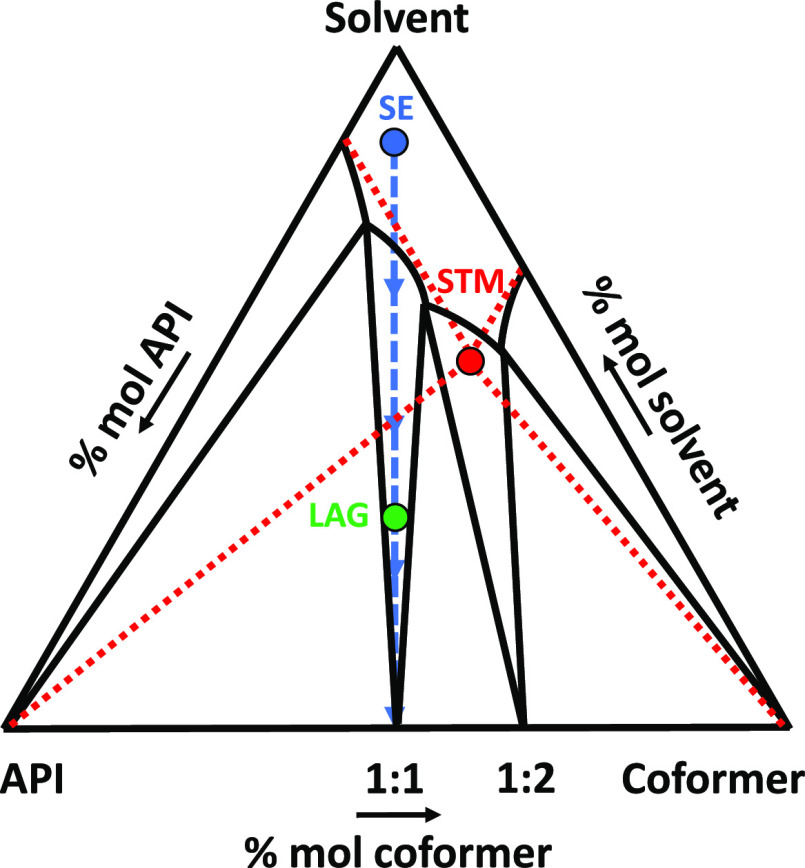
Schematic isothermal ternary phase diagram describing a system forming a 1:1 and a 1:2 cocrystal with congruent solubilities. The red point is the theoretical eutectic composition between API and the coformer at that specific temperature (solution doubly saturated in API and the coformer, computed from pure component ideal solubilities) chosen as the screening composition for the STM method. The green point corresponds to an arbitrary stoichiometric ratio screened by the LAG method. The blue dashed line corresponds to the crystallization pathway with the SE method from a stoichiometric undersaturated solution (blue point).

The stacked bar chart in Figure 11 gives a quantified overview of the ratio of positive (light blue), negative (red), and unscreenable (gray) responses of coformers for the methods and their number of new cocrystals identified during our screening. LAG allowed to cover the largest response on coformer ability to form cocrystals with PZQ, whether it is positive or negative, thereby showing the largest screenable coformer fraction (parameter *R*_1_ = 90%). The coformers that could not be screened with LAG were due to formation of amorphous phases because of LAG’s high energetic process. This limitation of LAG could be explained by the molecule mobility being too low to crystallize from an intermediate amorphous state in such conditions, because of a glass transition temperature being above the experiment temperature. Coformer success rate parameter *R*_2_ for LAG indicates that 41% of screened coformers resulted in a positive response to cocrystallization. Multiple new forms for one coformer were obtained by changing the solvent, allowing to find cocrystals with different stoichiometries or cocrystal solvates (coformer pluriformity parameter *R*_3_ = 18%). This demonstrates the high versatility of LAG as it does not require solubilization of the solid material, the solvent acting only as a catalytic medium and therefore a simplified solvent screening. For this reason, the cocrystal with orcinol (**29**) is specific to LAG as the coformer had solvent incompatibility with other methods, while LAG did not have this limitation. With the cocrystal(s) stability domain(s) in the ternary phase diagrams depending on pure component solubilities, using various solvents and especially ones in which solubility ratios between the coformer and API are different proves to be a conclusive strategy for optimal screening with LAG. The method also permits freedom regarding the compositions that can be screened (position of green dot in Figure 12 and Figure 13 is not fixed) and, therefore, multiple trials to access experimentally phase diagram domains where a cocrystal is stable. In this study, we limited ourselves to equimolar mixtures, but trying different ratios to investigate in-depth systems likely to present multiple forms would be relevant. With a quick experiment time (30 min), the accessibility of ball mill equipment, and low material consumption, LAG is confirmed to be highly convenient and ideal for quick and efficient screening. The acquisition of only phases suspected to be stable here with LAG is interesting to highlight, as despite constraining dynamic conditions for the system with a highly energetic milling, it always reached thermodynamic equilibrium. With a total of 13 new phases suspected to be stable that are identified with LAG, its new stable cocrystal coverage parameter *R*_4_ of 76% is the highest.

From Figure 11, the SE method presents the lowest number of conclusive responses on the coformer ability to cocrystallize, with the smallest screenable coformer fraction parameter *R*_1_ of 60%. This is mainly due to solvent incompatibility with the coformers. SE indeed requires quick solvent screening to find solvents able to solubilize both coformers and being volatile under the screening conditions. However, additional limitations are observed during cocrystal preparation as many attempts resulted in amorphous/oil formation during evaporation. These experimental issues to reach thermodynamic equilibrium are system dependent and unpredictable, which makes SE uncertain and mainly based on trial and error. It can be explained by an evaporation rate difficult to control, inducing more easily the formation of amorphous mixtures and also metastable phases. Indeed, only SE gave cocrystals we concluded to be metastable, for a total of three, with pimelic acid (**4**), hydroquinone (**15**), and 2,4-dihydroxybenzoic acid (**28**). They are considered false positives in the context of a cocrystal screening campaign, and therefore such uncertainty is generally unwanted. Furthermore, a new cocrystal solvate with 4-aminosalicylic acid (**12**) and MeCN that is identified by LAG and STM is missed by SE (Figure 7). It indicates SE can also be unreliable, probably due to the pathway of its composition evolution during evaporation that can cause trouble if the cocrystal has a noncongruent solubility in a solvent (see Figure 12). Indeed, in this example, the first solid phase to crystallize is a pure coformer that could continue to crystallize out of equilibrium if the cocrystal is not kinetically favored, possibly skipping the apparition of the latter, especially at the end of the SE experiment in which the last solvent evaporates as there are large supersaturations and risk of possible metastable forms crystallizing out. However, the coformer success rate parameter *R*_2_ for SE is the highest with 56% of screened coformers positive to cocrystallization. It means positive cocrystallization experiments had less issues to give a final solid with SE than negative ones. It is possibly explained by cocrystals identified in our study having a lower energetic barrier to crystallize than pure coformer solids. Nevertheless, no generalities can be concluded because of the small amount of data. SE coformer pluriformity parameter *R*_3_ of 20% is similar to the LAG one, meaning trial and error changes of solvent allow to find different stoichiometries and solvation in cocrystals. SE is therefore a highly convenient method for quick screening, with only basic equipment required, short experiment time, and low material consumption. However, with only nine new phases found that are suspected to be stable, its new stable cocrystal coverage parameter *R*_4_ of 53% in our study is the smallest and highlights some uncertainty and unreliability.

STM method gave almost as much conclusive data as LAG, with a screenable coformer fraction parameter *R*_1_ of 83%. Despite its solvent-based nature, only five coformers could not be screened due to solvent incompatibility. However, STM is not the most convenient as it requires solvent screening and the acquisition of accurate solubility curves prior to cocrystal screening, which takes a long experimental time and consumes more material than LAG and SE. Moreover, Crystal16 or other specific equipment for solubility curve determination is necessary. STM was preferred to cooling crystallization of 1:1 molar ratio solutions that has the risk of missing new forms.^53^ Indeed, STM-screened compositions are favored thermodynamically compared to arbitrary compositions as they are computed from pure component solubilities (see Figures 12 and13, red points). Throughout the cooling process, the composition equilibrates in the cocrystal stability domain due to a controlled low cooling rate and a final isothermal step. The method also guarantees the stable nature of new forms identified based on the thermodynamic principle “the less soluble, the more stable the solid phase.” The coformer success rate parameter *R*_2_ for STM is smaller (36%) than those for LAG and SE. The reason is the miss of the cocrystal hydrate with salicylic acid (**5**), found with LAG and SE. It could not be obtained from STM as contamination with water is not possible when using dry solvents, contrary to LAG and SE where the sample was in contact with ambient humidity. An advantage of STM is that coformers giving no new cocrystals with the method are screened accurately multiple times with varying stoichiometries by changing the solvent. Therefore, the negative results are more conclusive on the inability of these coformers to form a cocrystal with PZQ. By finding two cocrystals suspected to be stable for the coformers vanillic acid (**20**), 2,5-dihydroxybenzoic acid (**22**), and 2,4-dihydroxybenzoic acid (**28**), STM coformer pluriformity parameter *R*_3_ is the highest with 33%. For vanillic acid (**20**), two cocrystals suspected to be stable with different stoichiometries are found, while for 2,5-dihydroxybenzoic acid (**22**), one is solvated and not the other. Nonetheless, the specific cocrystal identified with STM for 2,4-dihydroxybenzoic acid (**28**) has not been resolved, and so it is not known if it has a different stoichiometry than 1:1 or if it is a cocrystal solvate. These results highlight the efficiency with STM to use a set of solvents presenting different solubilization behaviors regarding the coformers. It induces large variation of the screened compositions that are nonequimolar while guaranteeing the equilibration in a stable cocrystallization solubility domain, allowing to find more easily nonequimolar cocrystals as illustrated in Figure 13. With a total of 12 new phases suspected to be stable that are identified with STM, its new stable cocrystal coverage parameter *R*_4_ of 71% is high and comparable to LAG.

In the web chart in Figure 10, the defined parameters *R*_1_, *R*_2_, *R*_3_, and *R*_4_ are plotted for comparison of LAG, SE, and STM methods. It appears that in our screening, LAG allowed to screen the most coformers and to cover the largest number of new stable cocrystals found, making it the most successful method. STM is a close second, presenting very similar results but showing more multiple cocrystals found per positive coformer with less successful coformers than LAG. Finally, SE presents the most atypical results as due to experimental constraints, the number of screenable coformers was lowered. SE presents a high success rate among screenable coformers, but this result is balanced by the small number of new stable cocrystals covered as several metastable cocrystals were obtained.

**Figure 14:**
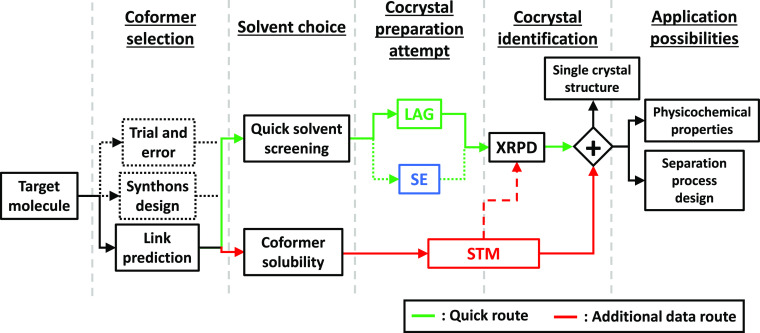
Workflow for cocrystal screening methodology advised from overall data obtained through PZQ cocrystal screening. Solid lines represent the pathways that should be preferred compared to dotted line ones that proved less efficient. Coformer selection results refer to the article from Devogelaer et al.^67^ on a link prediction approach for coformer selection applied to PZQ. LAG and SE are preparation methods only, ideal for a quick screening route (green), and require the combination with an identification method, XRPD being ideal. STM is a combination of preparation and identification methods, and XRPD identification step (dashed line), even if providing relevant additional information, can be skipped to go directly to solving crystal structure from single-crystal XRD information. STM is a slow screening method but allows to acquire additional solubility data (red). STM results also give phase diagram data useful for single-crystal growth and other applications.

Based on the results from our PZQ cocrystal screening campaign, we are therefore able to advise on the method selection strategy for screening optimization. Nevertheless, cocrystal screening is dependent on the studied thermodynamic systems, and it would be interesting to extend the comparison of the screening methods with the same parameters on a larger number of systems. Despite its high convenience, the SE method was weaker than LAG, and in a context of quick screening, we recommend using LAG rather than SE as represented in Figure 14. The results from LAG and STM are similar, although both methods differ a lot in their principles. STM method possesses a double status of cocrystal preparation and identification technique, directly giving a quantitative indication of cocrystal formation, while for LAG, the identification must be confirmed by a XRPD measurement. LAG is highly convenient for efficient results, which makes it a powerful method ideal for quick screening. However, no information is obtained regarding single-crystal growth possibilities or application possibilities. On the contrary, STM method is not convenient for quick screening as it requires longer experimental time, more material, and solubility curve determination work prior to screening. Nonetheless, it allows to acquire a large amount of accurate solubility data (see the Supporting Information) that can be collected in databases for future use. This is particularly relevant for pharmaceutical industry as the same pool of coformers are regularly used. Furthermore, when detecting a cocrystal with STM, experimental parameters to grow single crystals are also measured as stability domains of cocrystals are identified. The same compositions can therefore be used for slow cooling crystallization or temperature cycling growth experiments. These data can also be used later, for instance, to design a cocrystal production process.

## 5 Conclusions

Three common and accessible methods with different principles in their crystallization mechanism were investigated in a vast screening for cocrystals of PZQ. The methods were applied to PZQ with 30 coformers, which were identified based on a link prediction algorithm^67^ using data mining of the Cambridge Structural Database (CSD). A total of 17 cocrystals were identified, with 14 showing stability, and 12 new structures were resolved and reported.^67^ The large amount of data obtained in the screening helped to compare the efficiency of the cocrystal screening methods. LAG highlighted the best results, with the largest screenable coformer fraction (90%) and the highest number of cocrystals found that are suspected to be stable (13), even though amorphous phases are obtained in a few cases. SE showed numerous limitations due to its solvent dependence and its lack of crystallization control. Less coformers were screenable (60%), a lower number of cocrystals suspected to be stable was identified (9), three metastable phases were obtained, and an existing cocrystal was missed. STM method presented results as satisfactory as LAG. Less coformers were screenable (83%), but a similar number of cocrystals suspected to be stable was detected (12), revealing a tendency to identify multiple cocrystals per successful coformers. However, STM is less convenient than LAG and SE because of time and material required with solvent screening and solubility curve measurements. In summary, we advise LAG method for a quick and efficient screening route and STM for a slower route that provides relevant solubility data useful for future screenings, single-crystal growth, and eventual future cocrystal production in larger scale.

## Supporting Information Available

Materials, experimental methods and conditions, powder diffractograms for new cocrystals, solubility data of coformers, and saturation temperature results of coformer mixtures (PDF)

## Notes

Data availability statement; All data underpinning this publication are openly available from the University of Strathclyde KnowledgeBase at: https://doi.org/10.15129/501763a1-4a33-4a3e-999a-84faba625cd0.
