# Supplementary material for: Comparing and Quantifying the Efficiency of Cocrystal Screening Methods for Praziquantel
Source: Cryst Growth Des. 2022 Aug 25;22(9):5511–25. doi: 10.1021/acs.cgd.2c00615 (PMC9460446; doi:10.1021/acs.cgd.2c00615)
Supplement: Supplementary file 1 — cg2c00615_si_001.pdf [file cg2c00615_si_001.pdf]

Supporting Information: "Comparing and quantifying the efficiency of cocrystal screening methods for Praziquantel"

Maxime D. Charpentier[1], Jan-Joris Devogelaer[2], Arnoud Tijink[2], Hugo Meekes[2], Paul Tinnemans[2], Elias Vlieg[2], René de Gelder[2], Karen Johnston[3], Joop H. ter Horst[1,4]

1. EPSRC Centre for Innovative Manufacturing in Continuous Manufacturing and Crystallization (CMAC), University of Strathclyde, Technology and Innovation Centre, 99 George Street, Glasgow G1 1RD, U.K.

2. Radboud University, Institute for Molecules and Materials, Heyendaalseweg 135, 6525AJ Nijmegen, The Netherlands

3. Department of Chemical and Process Engineering, University of Strathclyde, James Weir Building, 75 Montrose Street, Glasgow G1 1XJ, U.K.

4. Laboratoire Sciences et Méthodes Séparatives, Université de Rouen Normandie, Place Emile Blondel, 76821 Mont Saint Aignan Cedex, France

Table of contents

S1 – Materials 2
S2 – Experimental conditions and XRPD results for LAG, SE, and STM methods 5
S3 – Experimental solubility results for STM screening method 11

S1 – Materials

This section is an overview of the coformers list used for the cocrystal screening, with their molecular structures (Figure S1), the suppliers and purities of materials used for LAG and SE methods (Table S1) and the ones used for STM method (Table S2).

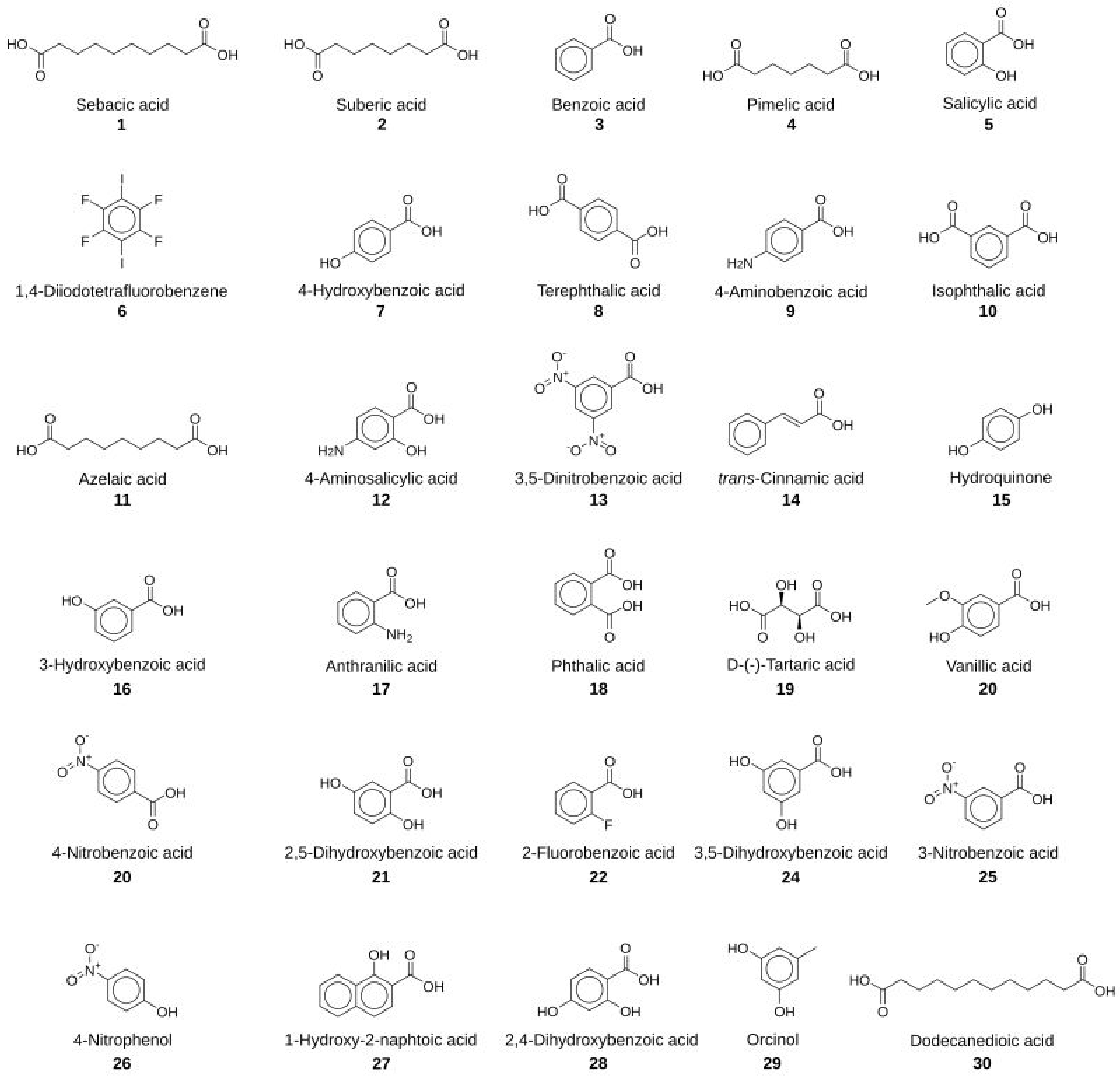

Figure S1: The thirty coformers screened experimentally for cocrystallization. Reprinted with permission from Crystal Growth & Design 2021 21 (6), 3428-3437. Copyright 2021 American Chemical Society.

Table S1: Coformers used for LAG and SE experiments with their CAS-number, chemical supplier, and purity. Reprinted with permission from Crystal Growth & Design 2021 21 (6), 3428-3437. Copyright 2021 American Chemical Society.

Rank	Coformer	CAS	Supplier	Purity
**1**	Sebacic acid	111-20-6	Acros Organics	98%
**2**	Suberic acid	505-48-6	Aldrich	98%
**3**	Benzoic acid	65-85-0	Sigma-Aldrich	≥ 99.5%
**4**	Pimelic acid	111-16-0	Sigma-Aldrich	98%
**5**	Salicylic acid	69-72-7	Alfa-Aesar	99%
**6**	1,4-Diiodotetrafluorobenzene	392-57-4	Merck	98%
**7**	4-Hydroxybenzoic acid	99-96-7	Fluka	≥ 99.8%
**8**	Terephthalic acid	100-21-0	Acros organics	≥ 99%
**9**	4-Aminobenzoic acid	150-13-0	Sigma-Aldrich	≥ 99%
**10**	Isophthalic acid	121-91-5	Aldrich	99%
**11**	Azelaic acid	123-99-9	Sigma-Aldrich	98%
**12**	4-Aminosalicylic acid	65-49-6	TCI Chemicals	≥ 98%
**13**	3,5-Dinitrobenzoic acid	99-34-3	Aldrich	97%
**14**	*trans*-Cinnamic acid	140-10-3	TCI Chemicals	> 98%
**15**	Hydroquinone	123-31-9	Merck	> 99%
**16**	3-Hydroxybenzoic acid	99-06-9	Aldrich	99%
**17**	Anthranilic acid	118-92-3	Sigma-Aldrich	> 98%
**18**	Phthalic acid	88-99-3	Merck	≥ 99.5%
**19**	D-(-)-Tartaric acid	147-71-7	Alfa-Aesar	99%
**20**	Vanillic acid	121-34-6	Sigma-Aldrich	≥ 97%
**21**	4-Nitrobenzoic acid	62-23-7	Fluorochem	99%
**22**	2,5-Dihydroxybenzoic acid	490-79-9	Fluorochem	99%
**23**	2-Fluorobenzoic acid	456-22-4	Merck	97%
**24**	3,5-Dihydroxybenzoic acid	99-10-5	Fluorochem	recryst. from MeCN
**25**	3-Nitrobenzoic acid	121-92-6	Sigma-Aldrich	99%
**26**	4-Nitrophenol	100-02-7	Acros Organics	99%
**27**	1-Hydroxy-2-naphtoic acid	86-48-6	Aldrich	≥ 97%
**28**	2,4-Dihydroxybenzoic acid	89-86-1	Aldrich	97%
**29**	Orcinol	504-15-4	Sigma-Aldrich	97%
**30**	Dodecanedioic acid	693-23-2	Acros Organics	99%

Table S2: Coformers used for STM experiments with their CAS-number, chemical supplier, and purity.

Rank	Coformer	CAS	Supplier	Purity
**1**	Sebacic acid	111-20-6	Sigma-Aldrich	99%
**2**	Suberic acid	505-48-6	Sigma-Aldrich	98%
**3**	Benzoic acid	65-85-0	Sigma-Aldrich	≥ 99.5%
**4**	Pimelic acid	111-16-0	Sigma-Aldrich	98%
**5**	Salicylic acid	69-72-7	Sigma-Aldrich	≥ 99%
**6**	1,4-Diiodotetrafluorobenzene	392-57-4	Sigma-Aldrich	98%
**7**	4-Hydroxybenzoic acid	99-96-7	Sigma-Aldrich	≥ 99%
**8**	Terephthalic acid	100-21-0	Sigma-Aldrich	98%
**9**	4-Aminobenzoic acid	150-13-0	Sigma-Aldrich	≥ 99%
**10**	Isophthalic acid	121-91-5	Sigma-Aldrich	99%
**11**	Azelaic acid	123-99-9	Sigma-Aldrich	98%
**12**	4-Aminosalicylic acid	65-49-6	Sigma-Aldrich	99%
**13**	3,5-Dinitrobenzoic acid	99-34-3	Sigma-Aldrich	99%
**14**	*trans*-Cinnamic acid	140-10-3	Sigma-Aldrich	99%
**15**	Hydroquinone	123-31-9	Sigma-Aldrich	≥ 99.5%
**16**	3-Hydroxybenzoic acid	99-06-9	Sigma-Aldrich	99%
**17**	Anthranilic acid	118-92-3	Sigma-Aldrich	≥ 99.5%
**18**	Phthalic acid	88-99-3	Sigma-Aldrich	≥ 99.5%
**19**	D-(-)-Tartaric acid	147-71-7	Sigma-Aldrich	99%
**20**	Vanillic acid	121-34-6	Sigma-Aldrich	≥ 97%
**21**	4-Nitrobenzoic acid	62-23-7	Sigma-Aldrich	98%
**22**	2,5-Dihydroxybenzoic acid	490-79-9	Sigma-Aldrich	98%
**23**	2-Fluorobenzoic acid	456-22-4	Sigma-Aldrich	97%
**24**	3,5-Dihydroxybenzoic acid	99-10-5	Sigma-Aldrich	97%
**25**	3-Nitrobenzoic acid	121-92-6	Sigma-Aldrich	99%
**26**	4-Nitrophenol	100-02-7	Sigma-Aldrich	≥ 99%
**27**	1-Hydroxy-2-naphtoic acid	86-48-6	Sigma-Aldrich	99%
**28**	2,4-Dihydroxybenzoic acid	89-86-1	Sigma-Aldrich	97%
**29**	Orcinol	504-15-4	Sigma-Aldrich	97%
**30**	Dodecanedioic acid	693-23-2	Sigma-Aldrich	99%

S2 – Experimental conditions and XRPD results for LAG, SE, and STM methods

This section is an overview of the experimental conditions used for LAG, SE, and STM (Table S3) and the corresponding XRPD patterns obtained in cases where new patterns emerged (Figures S2 to S16).

Table S3: Solvents used for the LAG, SE and STM screening methods. The stoichiometric ratios in parentheses are expressed as 'mol coformer per mol of PZQ' for the STM method. For LAG and SE, this ratio is always equal to 1.
'-[i]': symbol used when coformers present solubility issues in all solvents tried.
Adapted with permission from Crystal Growth & Design 2021 21 (6), 3428-3437. Copyright *2021* American Chemical Society.

Rank	Coformer	Solvent LAG	Solvent SE	Solvent STM (Ratio $mol_{coformer}$ per mol_{PZQ})	XRPD Figure associated
**1**	Sebacic acid	MeOH, EtOH	MeOH, EtOH, iPrOH	EtOH (1.4), AcOEt (0.3)	
**2**	Suberic acid	MeOH, EtOH	MeOH, EtOH	EtOH (2.1)	
**3**	Benzoic acid	EtOH	EtOH	EtOH (13.3), MeCN (3.3), AcOEt (12.9)	
**4**	Pimelic acid	MeCN	MeCN	MeCN (0.7), AcOEt (2.4)	S2
**5**	Salicylic acid	EtOH	EtOH	EtOH (10.6), MeCN (2.2), AcOEt (9.2)	S3
**6**	1,4-Diiodotetrafluorobenzene	MeCN	MeCN	EtOH (5)	S4
**7**	4-Hydroxybenzoic acid	MeCN	MeCN	EtOH (8.3), MeCN (0.9)	S5
**8**	Terephthalic acid	MeCN	-[i]	-[i]	
**9**	4-Aminobenzoic acid	MeCN	MeCN	EtOH (3.3), MeCN (1.5), AcOEt (3.4)	
**10**	Isophthalic acid	MeCN	-[i]	-[i]	
**11**	Azelaic acid	EtOH	EtOH	EtOH (4.1), AcOEt (0.8)	
**12**	4-Aminosalicylic acid	MeCN	MeCN	MeCN (0.4)	S6
**13**	3,5-Dinitrobenzoic acid	MeCN	MeCN	MeCN (0.9)	S7
**14**	*trans*-Cinnamic acid	MeCN	MeCN	EtOH (5.4), MeCN (1.4), AcOEt (5.5)	
**15**	Hydroquinone	MeCN	MeCN	MeCN (3.6)	S8
**16**	3-Hydroxybenzoic acid	EtOH	EtOH	EtOH (7.7 and 3.9), MeCN (0.9 and 0.8), AcOEt (4.2 and 3.4)	
**17**	Anthranilic acid	MeCN, MeOH	MeCN, MeOH	EtOH (6.2), MeCN (3), AcOEt (8.7)	
**18**	Phthalic acid	MeCN	-[i]	-[i]	
**19**	D-(-)-Tartaric acid	MeCN	MeCN	-[i]	
**20**	Vanillic acid	EtOH, MeCN	EtOH, MeCN	EtOH (2), MeCN (0.2), AcOEt (0.6)	S9, S10
**21**	4-Nitrobenzoic acid	MeCN	MeCN	EtOH (0.6), AcOEt (0.6)	
**22**	2,5-Dihydroxybenzoic acid	Acetone, MeCN	Acetone, MeCN	EtOH (9 and 4.5), MeCN (1.4), AcOEt (4.1)	S11, S12

Table S3 (continued). Adapted with permission from Crystal Growth & Design 2021 21 (6), 3428-3437. Copyright *2021* American Chemical Society.

Rank	Coformer	Solvent LAG	Solvent SE	Solvent STM (Ratio $mol_{coformer}$ per mol_{PZQ})	XRPD Figure associated
**23**	2-Fluorobenzoic acid	EtOH	EtOH	EtOH (17.4), MeCN (5.3), AcOEt (12.8)	
**24**	3,5-Dihydroxybenzoic acid	MeCN	MeCN	MeCN (0.7)	S13
**25**	3-Nitrobenzoic acid	EtOH	EtOH	MeCN (3.6), AcOEt (9.7)	
**26**	4-Nitrophenol	EtOH, MeCN	EtOH	EtOH (32.1)	
**27**	1-Hydroxy-2-naphtoic acid	MeCN	MeCN	AcOEt (0.6)	
**28**	2,4-Dihydroxybenzoic acid	MeCN	MeCN	EtOH (8.1), MeCN (1), AcOEt (5.7 and 4.3)	S14, S15
**29**	Orcinol	EtOH, MeCN	EtOH, MeCN	-[i]	S16
**30**	Dodecanedioic acid	MeCN	EtOH	EtOH (0.9)	

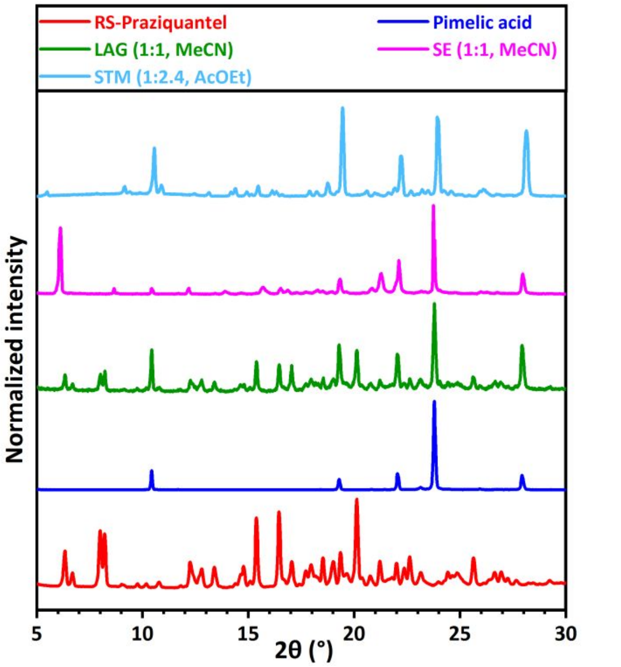

Figure S2: XRPD patterns for RS-PZQ, pimelic acid and solid phases obtained from their mixtures after LAG, SE and STM (with corresponding solvent and molar ratio between coformer and PZQ $M_{PZQ:cof}$). New peaks are identified for SE (structure could not be resolved), while LAG and STM resulted in a physical mixtures of the coformers.

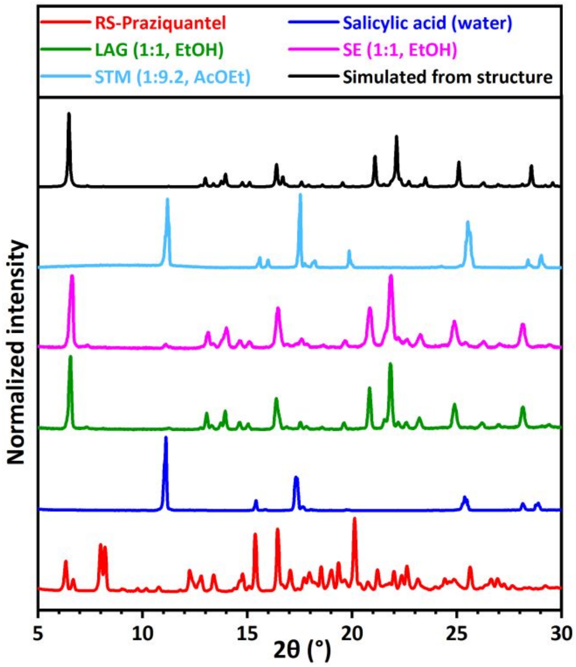

Figure S3: XRPD patterns for RS-PZQ, salicylic acid and solid phases obtained from their mixtures after LAG, SE and STM (with corresponding solvent and molar ratio between coformer and PZQ $M_{PZQ:cof}$). The simulated powder pattern from resolved cocrystal hydrate (CCDC 2054486) is added for comparison. This new pattern is identified for LAG and SE while STM resulted in a physical mixture in solvents tried.

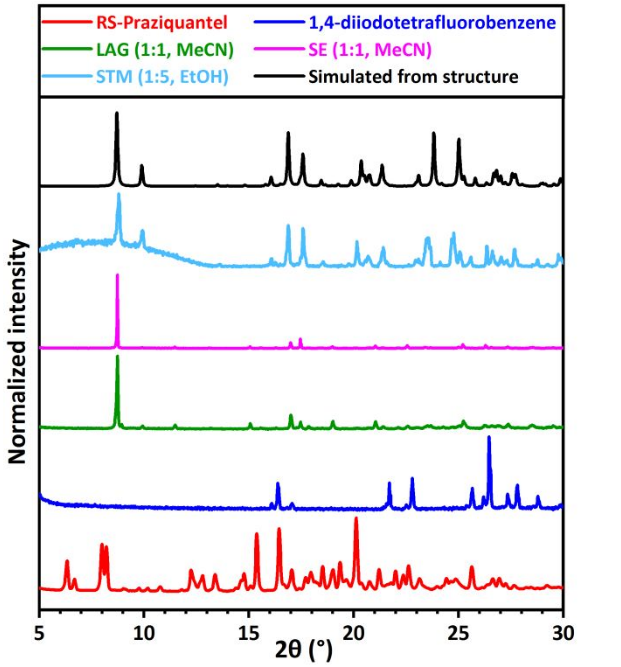

Figure S4: XRPD patterns for RS-PZQ, 1,4-diiodotetrafluorobenzene and solid phases obtained from their mixtures after LAG, SE and STM (with corresponding solvent and molar ratio between coformer and PZQ $M_{PZQ:cof}$). The simulated powder pattern from resolved cocrystal (CCDC 2054495) is added for comparison. This new pattern is identified for LAG, SE and STM.

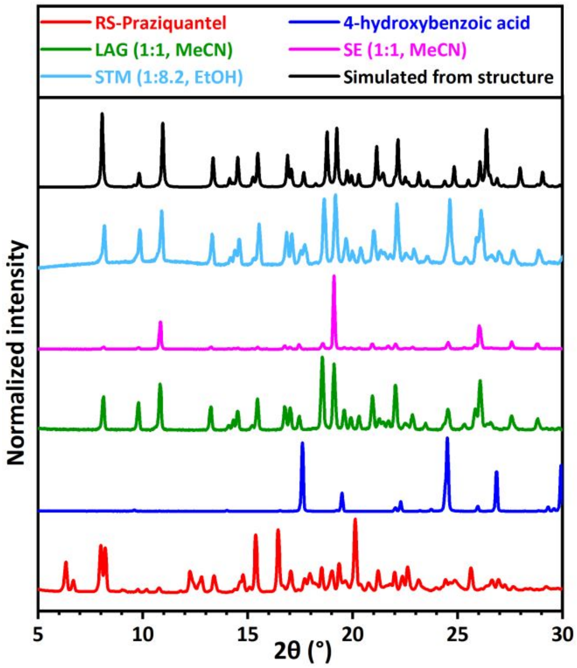

Figure S5: XRPD patterns for RS-PZQ, 4-hydroxybenzoic acid and solid phases obtained from their mixtures after LAG, SE and STM (with corresponding solvent and molar ratio between coformer and PZQ $M_{PZQ:cof}$). The simulated powder pattern from resolved cocrystal (CCDC 2054492) is added for comparison. This new pattern is identified for LAG, SE and STM.

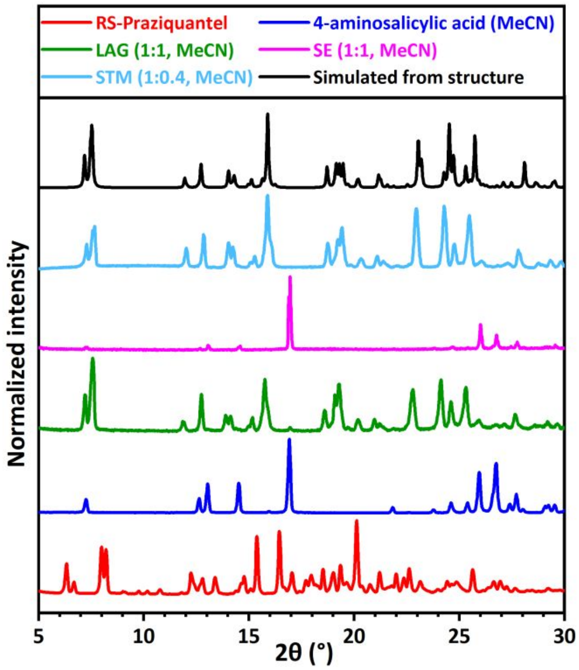

Figure S6: XRPD patterns for RS-PZQ, 4-aminosalicylic acid and solid phases obtained from their mixtures after LAG, SE and STM (with corresponding solvent and molar ratio between coformer and PZQ $M_{PZQ:cof}$). The simulated powder pattern from resolved cocrystal solvate (CCDC 2054493) is added for comparison. This new pattern is identified for LAG and STM, but not SE.

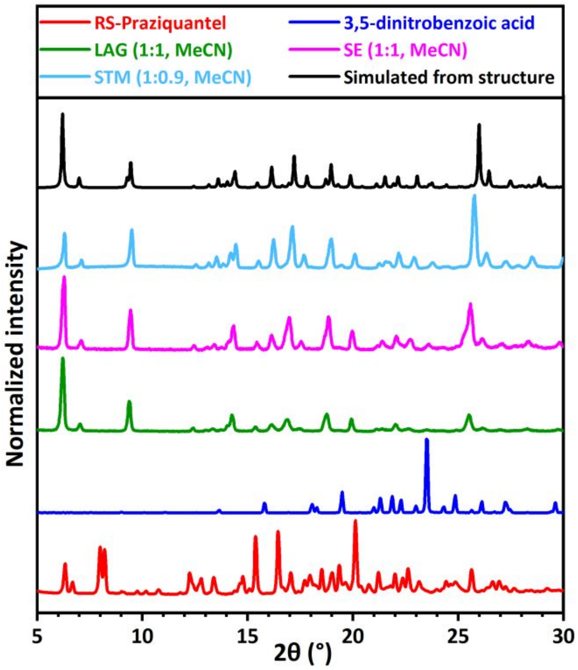

Figure S7: XRPD patterns for RS-PZQ, 3,5-dinitrobenzoic acid and solid phases obtained from their mixtures after LAG, SE and STM (with corresponding solvent and molar ratio between coformer and PZQ $M_{PZQ:cof}$). The simulated powder pattern from resolved cocrystal (CCDC 2054491) is added for comparison. This new pattern is identified for LAG, SE and STM.

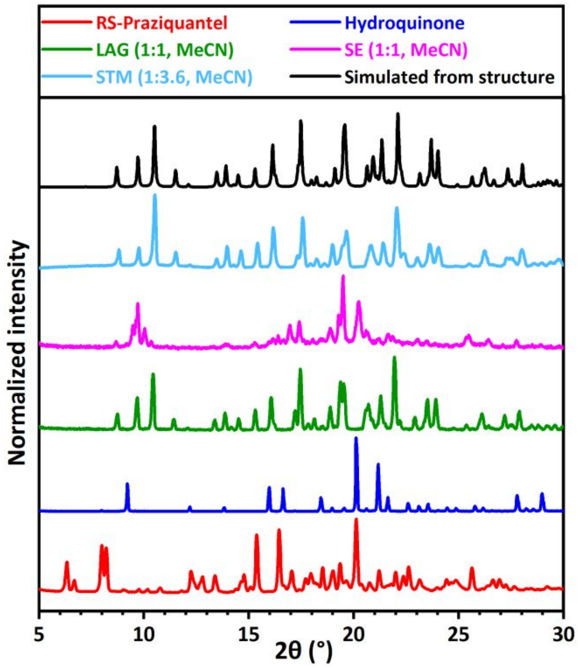

Figure S8: XRPD patterns for RS-PZQ, hydroquinone and solid phases obtained from their mixtures after LAG, SE and STM (with corresponding solvent and molar ratio between coformer and PZQ $M_{PZQ:cof}$). The simulated powder pattern from resolved cocrystal (CCDC 2054497) is added for comparison. This new pattern is identified for LAG and STM. SE presents a different new pattern (structure not resolved)

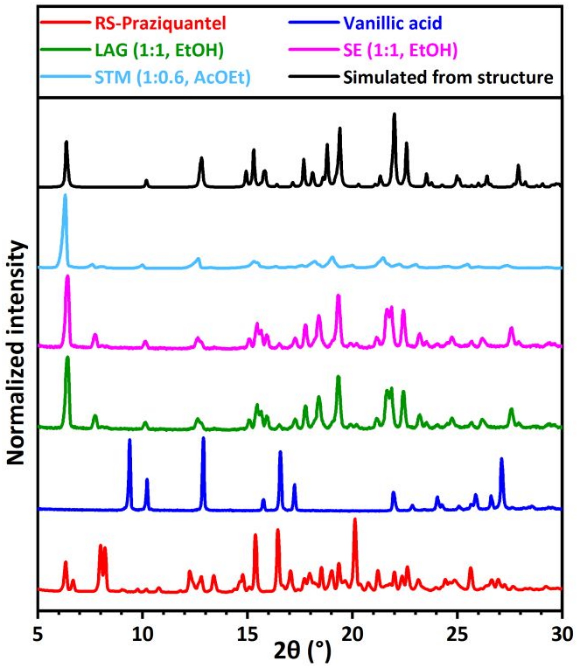

Figure S9: XRPD patterns for RS-PZQ, vanillic acid and solid phases obtained from their mixtures after LAG, SE and STM (with corresponding solvent and molar ratio between coformer and PZQ $M_{PZQ:cof}$). The simulated powder pattern from resolved cocrystal (CCDC 2054490) is added for comparison. This new pattern is identified for LAG, SE and STM.

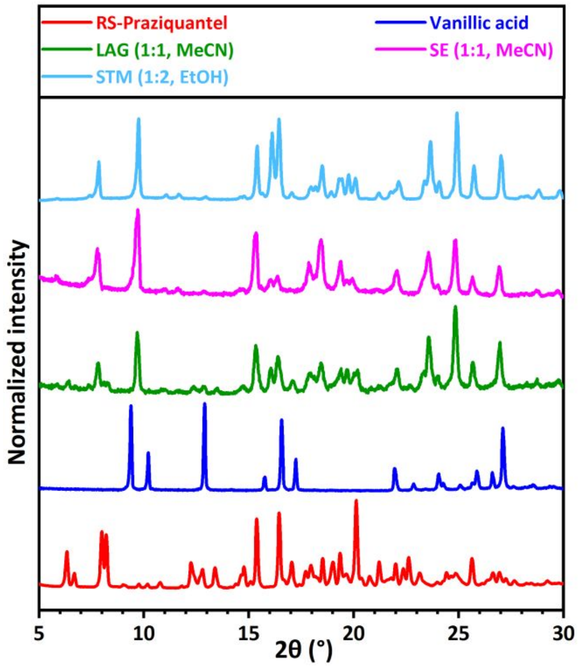

Figure S10: XRPD patterns for RS-PZQ, vanillic acid and solid phases obtained from their mixtures after LAG, SE and STM (with corresponding solvent and molar ratio molar ratio between coformer and PZQ $M_{PZQ:cof}$). A new pattern is identified for LAG, SE and STM. It differs from the resolved cocrystal (CCDC 2054490) (in Figure S8) also obtained with vanillic acid in other solvents.

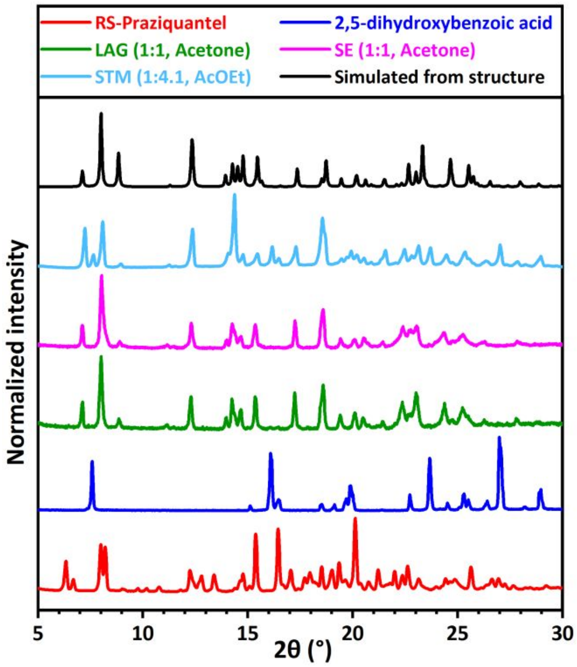

Figure S11: XRPD patterns for RS-PZQ, 2,5-dihydroxybenzoic acid and solid phases obtained from their mixtures after LAG, SE and STM (with corresponding solvent and molar ratio molar ratio between coformer and PZQ $M_{PZQ:cof}$). The simulated powder pattern from resolved cocrystal (CCDC 2054489) is added for comparison. This new pattern is identified for LAG, SE and STM.

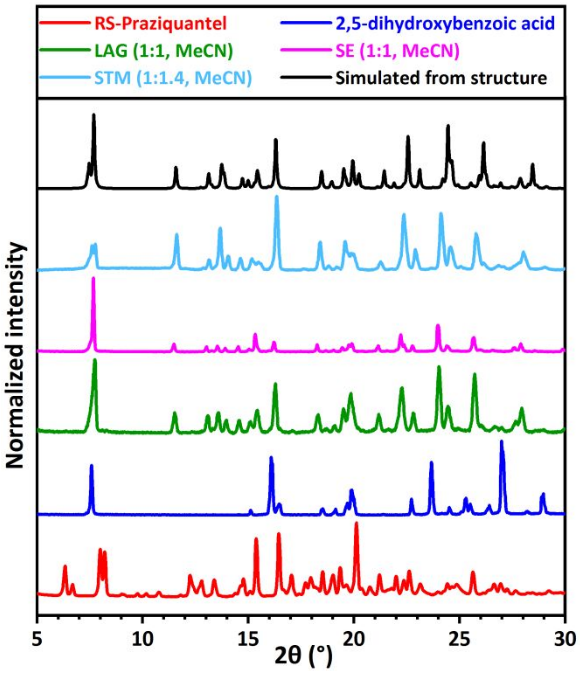

Figure S12: XRPD patterns for RS-PZQ, 2,5-dihydroxybenzoic acid and solid phases obtained from their mixtures after LAG, SE and STM (with corresponding solvent and molar ratio between coformer and PZQ $M_{PZQ:cof}$). The simulated powder pattern from resolved cocrystal solvate (CCDC 2054487) is added for comparison. This new pattern is identified for LAG, SE and STM.

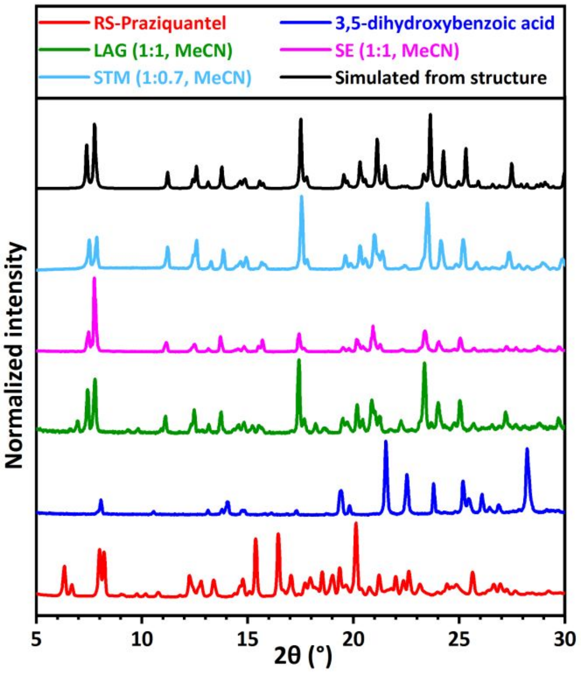

Figure S13: XRPD patterns for RS-PZQ, 3,5-dihydroxybenzoic acid and solid phases obtained from their mixtures after LAG, SE and STM (with corresponding solvent and molar ratio between coformer and PZQ $M_{PZQ:cof}$). The simulated powder pattern from resolved cocrystal solvate (CCDC 2054496) is added for comparison. This new pattern is identified for LAG, SE and STM.

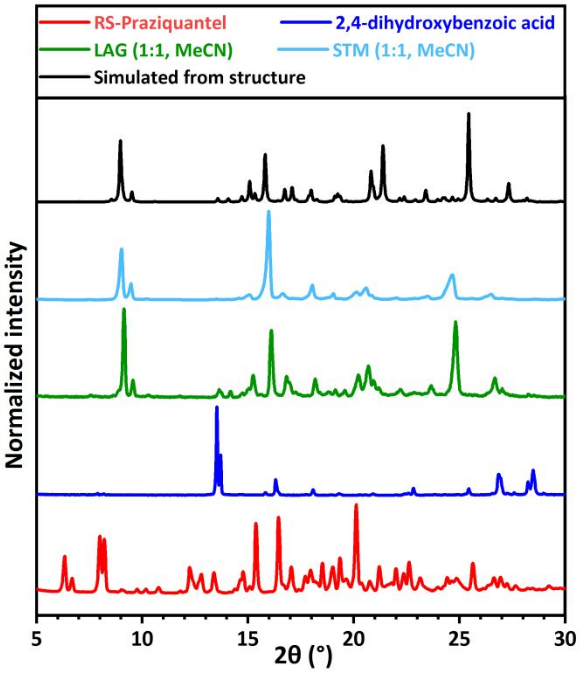

Figure S14: XRPD patterns for RS-PZQ, 2,4-dihydroxybenzoic acid and solid phases obtained from their mixtures after LAG and STM (with corresponding solvent and molar ratio between coformer and PZQ $M_{PZQ:cof}$). The simulated powder pattern from resolved cocrystal (CCDC 2054494) is added for comparison. This new pattern is identified for LAG and STM.

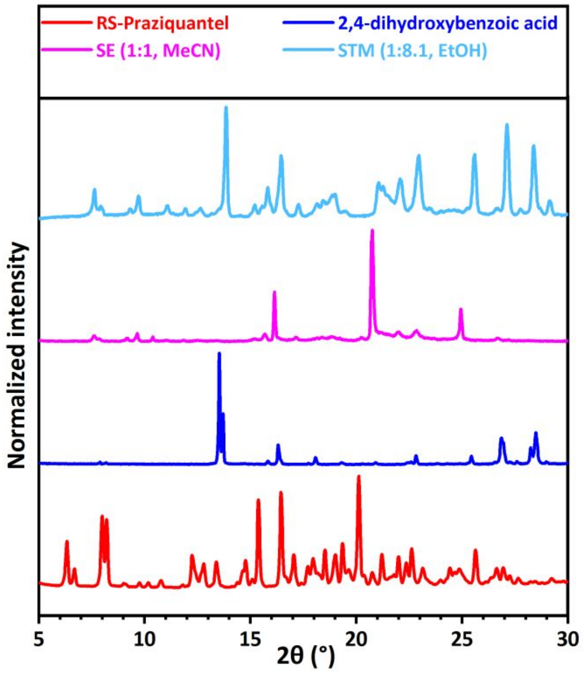

Figure S15: XRPD patterns for RS-PZQ, 2,4-dihydroxybenzoic acid and solid phases obtained from their mixtures after SE and STM (with corresponding solvent and molar ratio between coformer and PZQ $M_{PZQ:cof}$). New different patterns are identified for SE and STM. They differ from the resolved cocrystal (CCDC 2054494) (in Figure S14) also obtained with 2,4-dihydroxybenzoic in other conditions.

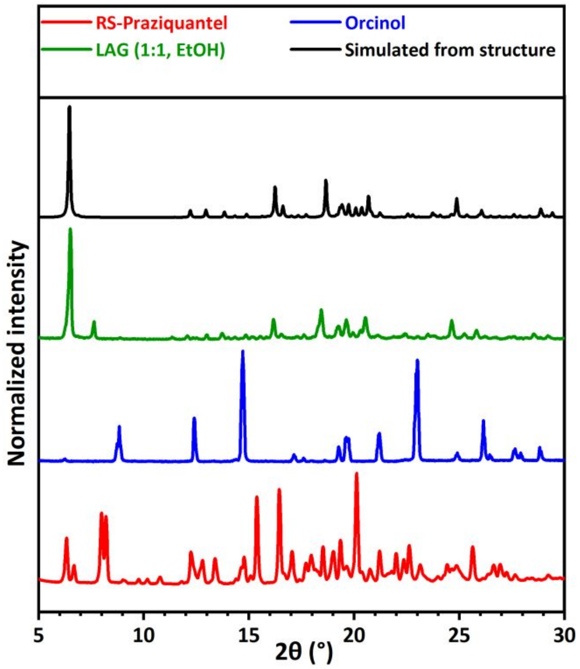

Figure S16: XRPD patterns for RS-PZQ, orcinol and solid phase obtained from a mixtures after LAG (with corresponding solvent and molar ratio between coformer and PZQ $M_{PZQ:cof}$). The simulated powder pattern from resolved cocrystal (CCDC 2054488) is added for comparison.

S3 – Experimental solubility results for STM screening method

This section presents the experimental temperature-solubility measurements data for RS-PZQ and pure coformers (Tables S4 to S29) and their associated Van 't Hoff plots (Figures S17 to S42) in ethanol (EtOH), acetonitrile (MeCN) and ethyl acetate (AcOEt). Table S30 summarizes STM method results with the ternary compositions screened and temperature differences ΔT measured for all coformers in solvents tried between reference temperature T_r and average experimental saturation temperature T_{sat}.

$$\ln(x^*) = -\frac{\Delta H^f}{R}\left(\frac{1}{T} - \frac{1}{T_m}\right) \qquad \text{Van 't Hoff equation}$$

x^*: solubility (molar fraction); ΔH^f: fusion enthalpy (J.mol^{-1}); T_m: melting temperature of the compound (K); T: temperature (K); R: universal gas constant equal to 8.314 J.mol^{-1}.K^{-1}

<u>Praziquantel (*RS*-PZQ)</u>

Table S4: *RS*-PZQ solubilities with Crystal16.

	Molar fraction x	Average T_{sat} (K)
EtOH	0.00825	289.15
	0.01106	296.05
	0.01511	301.92
	0.01823	304.95
	0.02156	307.88
	0.02506	310.48
	0.03036	313.52
	0.03598	316.55
MeCN	0.01234	297.25
	0.01609	303.12
	0.01877	306.25
	0.02271	310.78
	0.02537	313.05
	0.02879	315.28
	0.03370	318.18
	0.03806	320.75
AcOEt	0.1370	296.78
	0.1740	303.88
	0.2197	310.58
	0.2519	313.35
	0.3026	319.45
	0.3283	321.38
	0.3739	324.58
	0.4056	325.38

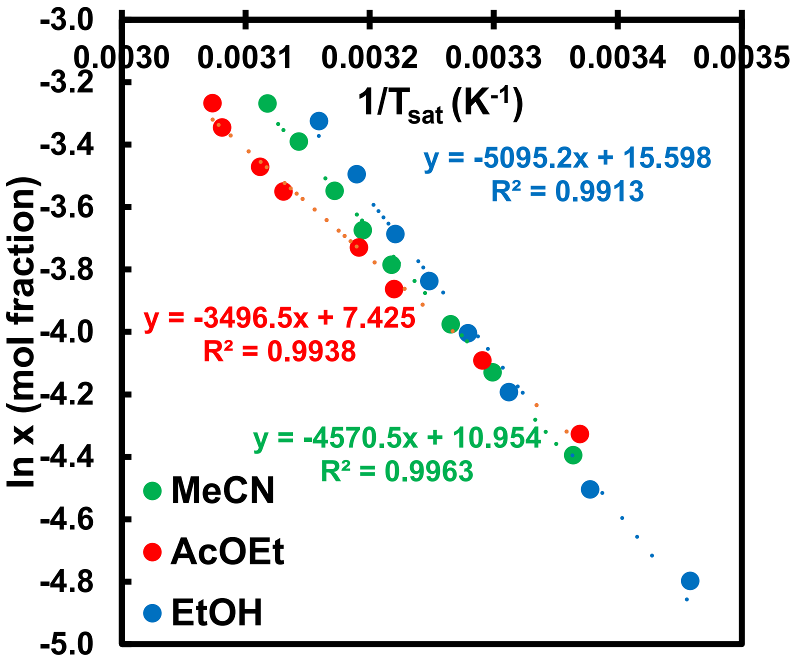

Figure S17: Van 't Hoff plots for RS-PZQ.

<u>Sebacic acid (**1**)</u>

Table S5: Sebacic acid solubilities with Crystal16.

	Molar fraction x	Average T_{sat} (K)
EtOH	0.03163	309.32
	0.04626	317.38
	0.05679	322.05
	0.06796	326.58
	0.02169	301.68
AcOEt	0.00356	315.25
	0.00438	317.82
	0.00537	319.98
	0.00139	296.78
	0.00180	301.05
	0.00224	305.48

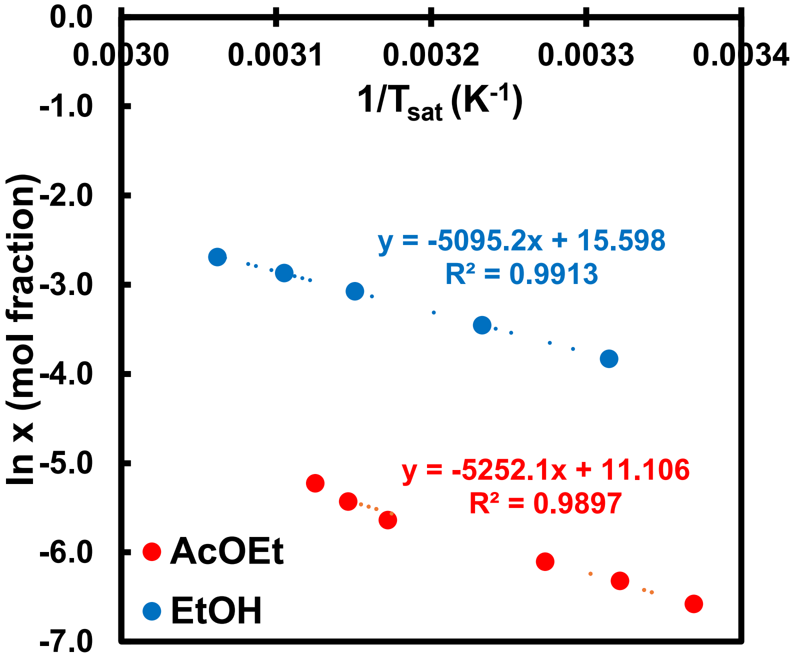

Figure S18: Van 't Hoff plots for sebacic acid.

<u>Suberic acid (**2**)</u>

Table S6: Suberic acid solubilities with Crystal16.

	Molar fraction x	Average T_{sat} (K)
EtOH	0.02919	299.22
	0.04384	309.12
	0.05640	315.92
	0.03887	305.65

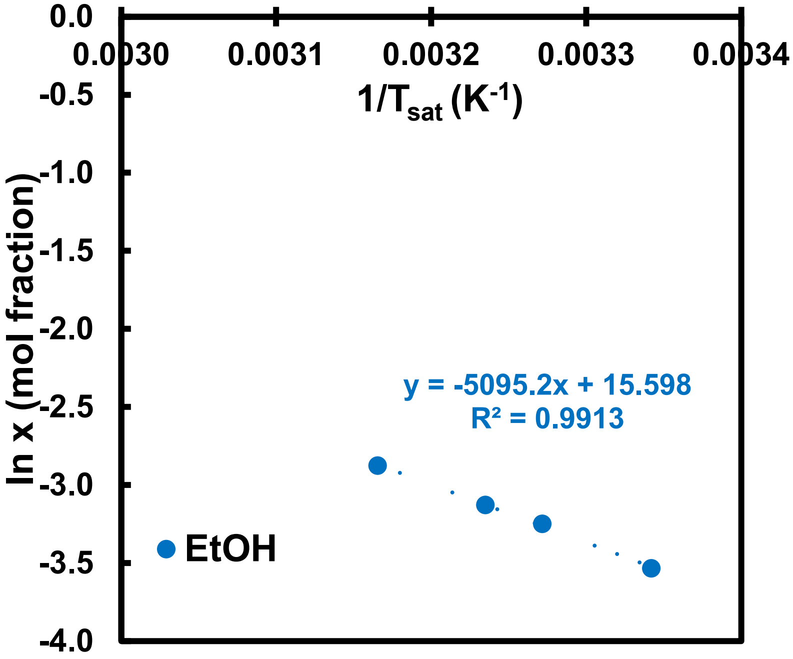

Figure S19: Van 't Hoff plot for suberic acid.

Benzoic acid (**3**)

Table S7: Benzoic acid solubilities with Crystal16.

	Molar fraction x	Average T_{sat} (K)
EtOH	0.14250	287.88
	0.16241	294.95
	0.18061	300.68
	0.19945	308.15
	0.21627	312.92
	0.23651	316.05
MeCN	0.03697	290.62
	0.04574	296.68
	0.05180	299.98
	0.06041	304.65
	0.07022	309.18
AcOEt	0.08590	294.58
	0.09826	299.85
	0.10996	304.45
	0.11754	307.42

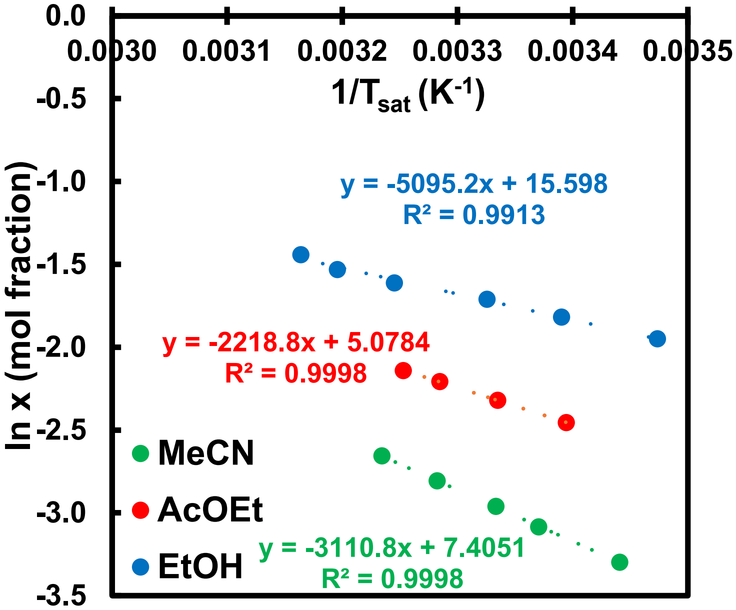

Figure S20: Van ’t Hoff plots for benzoic acid.

Pimelic acid (**4**)

Table S8: Pimelic acid solubilities with Crystal16.

	Molar fraction x	Average T_{sat} (K)
MeCN	0.01957	308.62
	0.02999	314.15
	0.04210	318.98
	0.05619	323.25
	0.00979	298.72
	0.01266	302.55
	0.01508	305.05
AcOEt	0.02286	307.52
	0.02750	310.78
	0.03027	312.18
	0.02061	304.72
	0.02379	307.75

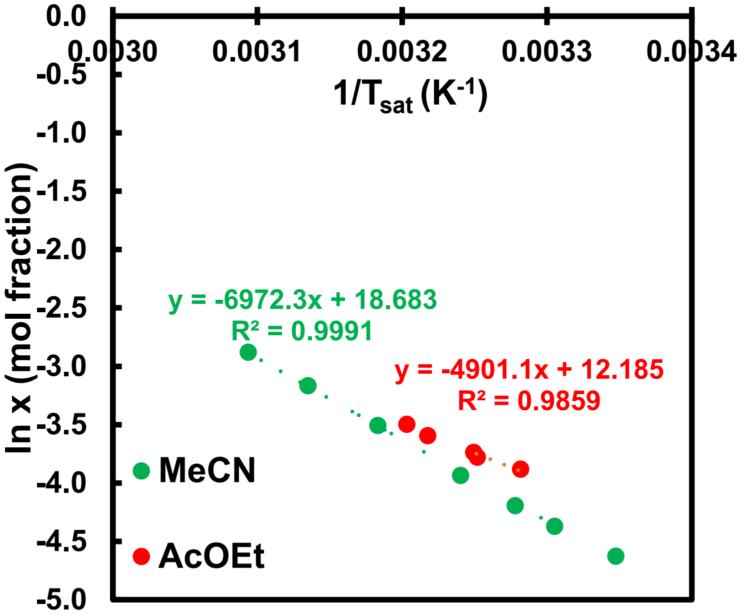

Figure S21: Van ’t Hoff plots for pimelic acid.

Salicylic acid (**5**)

Table S9: Salicylic acid solubilities with Crystal16.

	Molar fraction x	Average T_{sat} (K)
**EtOH**	0.12098	286.65
	0.15694	303.38
	0.14560	299.58
	0.17904	312.82
	0.19251	320.02
	0.20828	326.02
**MeCN**	0.04238	306.48
	0.05062	311.48
	0.05605	314.60
	0.06332	317.02
	0.02647	291.58
	0.03132	296.98
	0.03795	303.55
	0.07017	323.52
**AcOEt**	0.07077	294.72
	0.08518	306.05
	0.10858	322.32

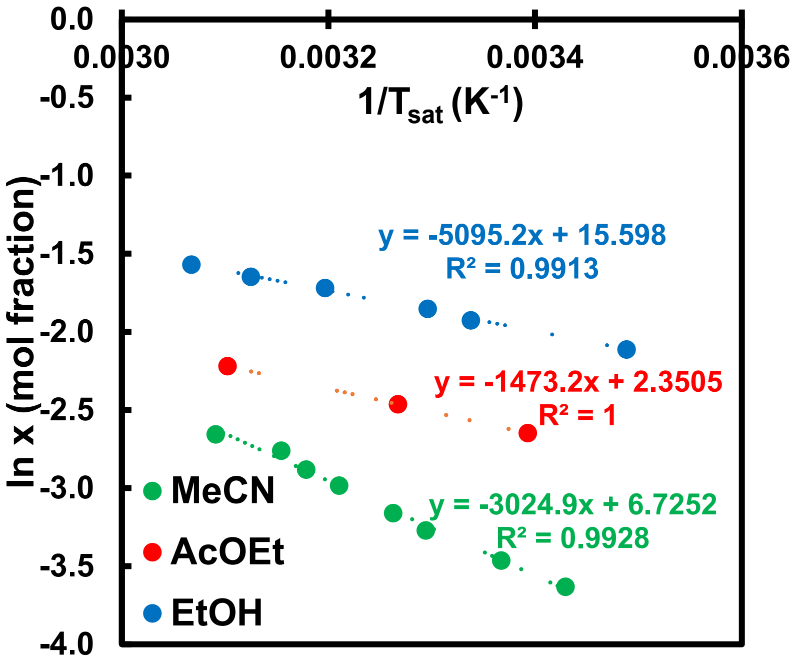

Figure S22: Van 't Hoff plots for salicylic acid.

1,4-Diiodotetrafluorobenzene (**6**)

Table S10: 1,4-Diiodotetrafluorobenzene solubilities with Crystal16.

	Molar fraction x	Average T_{sat} (K)
EtOH	0.07043	297.80
	0.08175	305.38
	0.09557	312.48
	0.07846	303.62
	0.11166	319.42

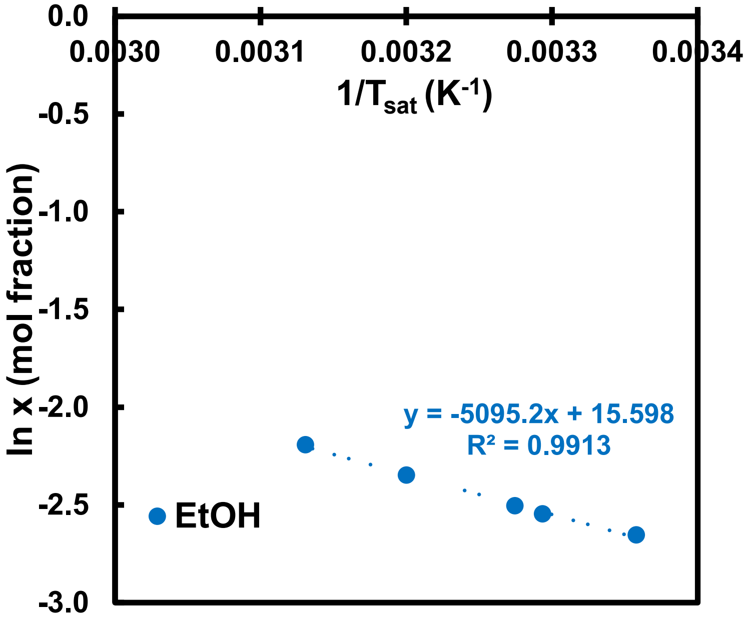

Figure S23: Van ’t Hoff plot for 1,4-diiodotetrafluorobenzene.

4-Hydroxybenzoic acid (**7**)

Table S11: 4-Hydroxybenzoic solubilities with Crystal16.

	Molar fraction x	Average T_{sat} (K)
EtOH	0.12369	296.45
	0.13577	309.15
	0.14859	319.22
	0.12389	296.88
	0.12869	301.92
	0.15166	323.32
MeCN	0.01581	298.55
	0.01877	304.38
	0.02170	309.02
	0.02391	312.52

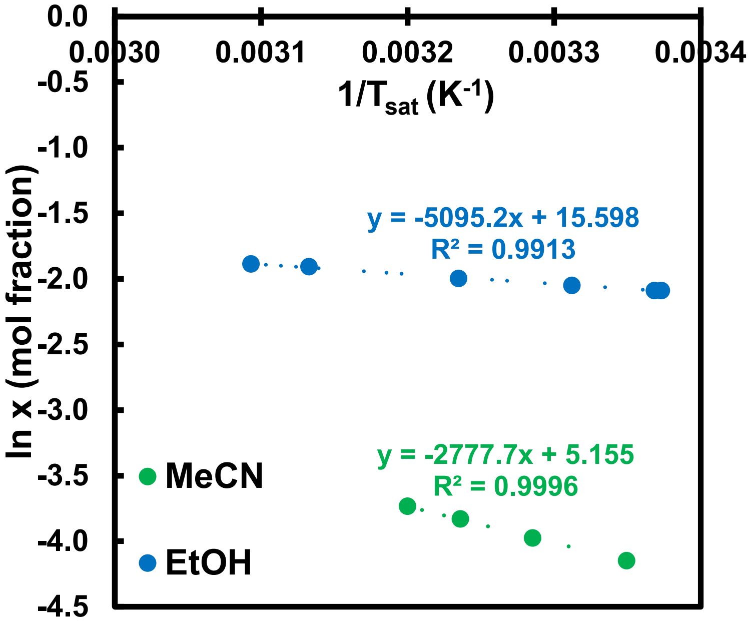

Figure S24: Van ’t Hoff plots for 4-hydroxybenzoic acid.

<u>4-aminobenzoic acid (**9**)</u>

Table S12: 4-aminobenzoic acid solubilities with Crystal16.

	Molar fraction x	Average T_{sat} (K)
EtOH	0.04672	295.12
	0.06511	314.02
	0.08930	327.55
	0.05931	310.25
	0.07762	323.45
MeCN	0.03040	306.78
	0.03548	311.35
	0.04367	315.22
	0.02094	294.42
	0.02385	299.22
	0.02892	305.88
AcOEt	0.02539	285.15
	0.02997	300.92
	0.03353	309.85
	0.03850	317.45

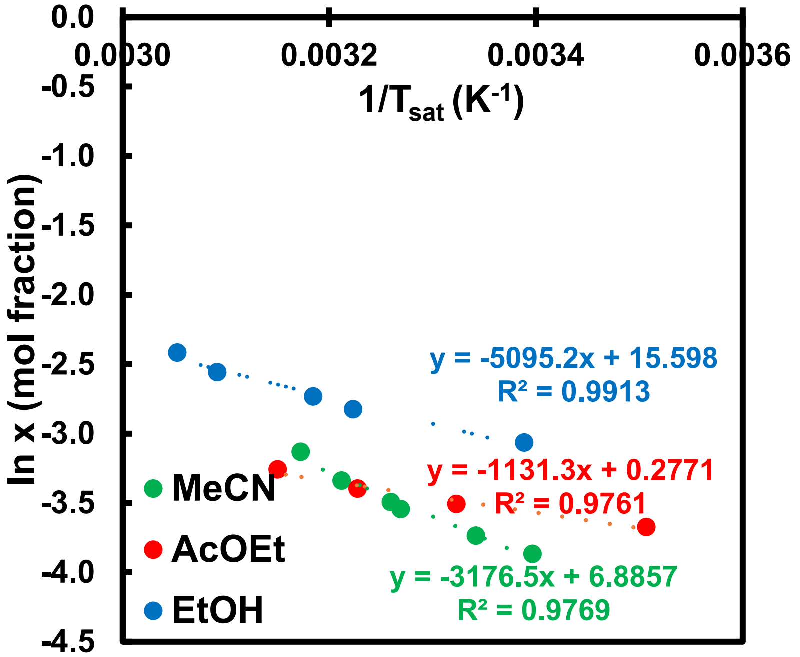

Figure S25: Van 't Hoff plots for 4-aminobenzoic acid.

<u>Azelaic acid (**11**)</u>

Table S13: Azelaic acid solubilities with Crystal16.

	Molar fraction x	Average T_{sat} (K)
EtOH	0.04803	294.58
	0.06705	302.18
	0.08730	311.22
	0.10216	317.38
AcOEt	0.00353	293.82
	0.00479	299.82
	0.00681	305.25
	0.00838	308.88

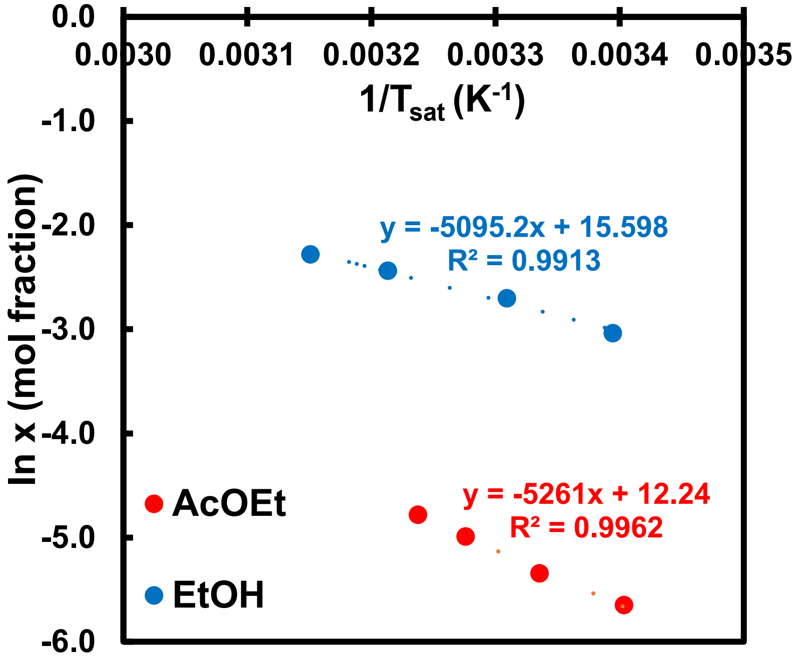

Figure S26: Van 't Hoff plots for azelaic acid.

4-Aminosalicylic acid (**12**)

Table S14: 4-Aminosalicylic acid solubilities with Crystal 16.

	Molar fraction x	Average T_{sat} (K)
**MeCN**	0.00824	311.02
	0.01064	320.08
	0.01270	329.15
	0.00532	294.22
	0.00690	304.52

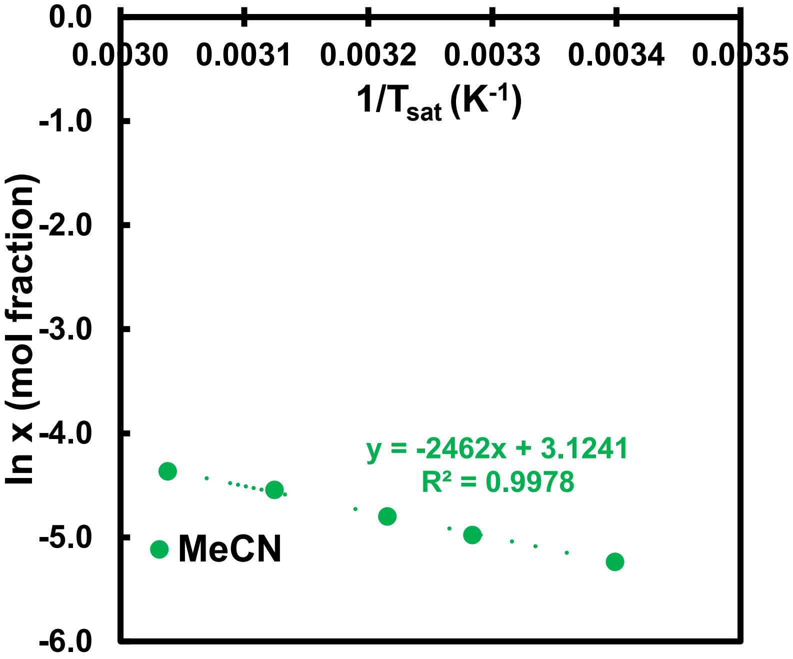

Figure S27: Van 't Hoff plots for 4-aminosalicylic acid.

3,5-dinitrobenzoic acid (**13**)

Table S15: 3,5-dinitrobenzoic acid solubilities with Crystal 16.

	Molar fraction x	Average T_{sat} (K)
**MeCN**	0.01662	304.55
	0.02308	315.85
	0.02787	322.38
	0.03295	328.52
	0.01124	291.55
	0.01400	298.48

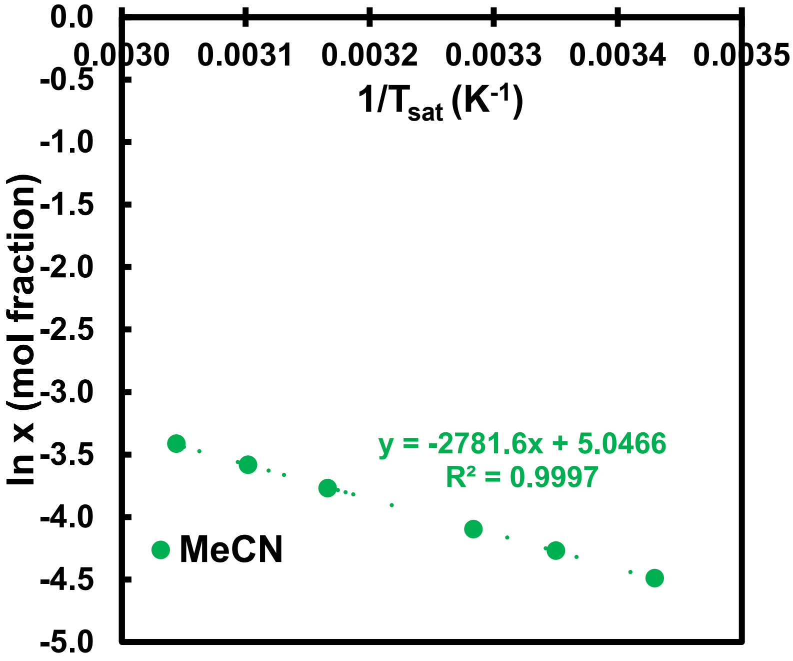

Figure S28: Van 't Hoff plots for 3,5-dinitrobenzoic acid.

trans-cinnamic acid (**14**)

Table S16: *trans*-cinnamic acid solubilities with Crystal16.

	Molar fraction x	Average T_{sat} (K)
**EtOH**	0.09441	307.55
	0.11460	320.15
	0.12712	323.62
	0.14219	330.32
**MeCN**	0.02600	304.58
	0.03285	310.65
	0.04132	316.05
	0.05008	320.05
	0.01819	296.18
	0.02275	301.78
**AcOEt**	0.03625	293.88
	0.04746	303.65
	0.05413	308.12
	0.06260	313.38

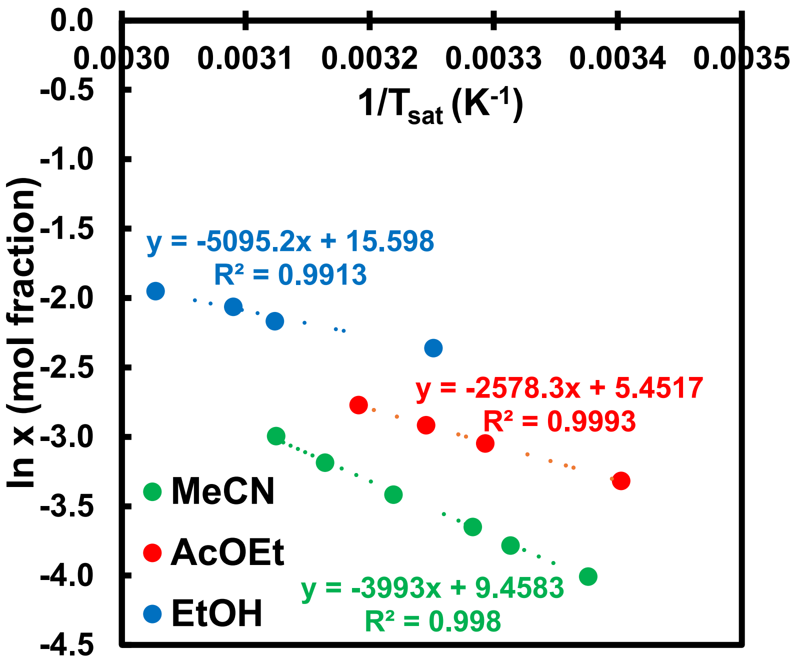

Figure S29: Van 't Hoff plots for *trans*-cinnamic acid.

Hydroquinone (**15**)

Table S17: Hydroquinone solubilities with Crystal 16.

	Molar fraction x	Average T_{sat} (K)
**MeCN**	0.06815	305.65
	0.08702	314.58
	0.10673	321.72
	0.12058	326.65
	0.05215	296.15
	0.03856	286.48

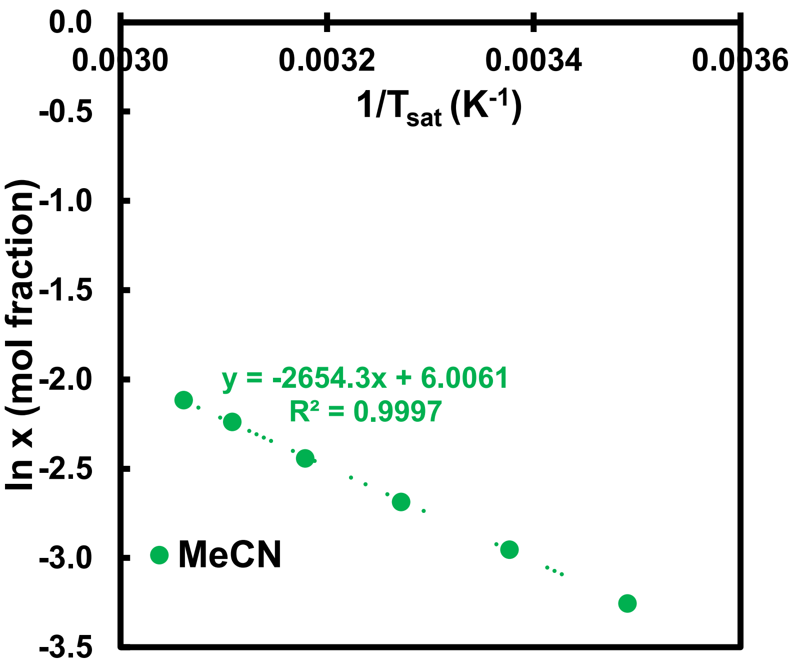

Figure S30: Van 't Hoff plots for hydroquinone.

3-hydroxybenzoic acid (**16**)

Table S18: 3-hydroxybenzoic acid solubilities with Crystal16.

	Molar fraction x	Average T_{sat} (K)
EtOH	0.11816	305.18
	0.14358	324.15
	0.11243	298.32
	0.12885	312.85
	0.13652	318.32
MeCN	0.01422	294.75
	0.01699	300.68
	0.01985	305.58
	0.02214	309.62
AcOEt	0.03386	296.87
	0.04548	314.55
	0.03857	303.18
	0.04221	308.28
	0.04867	317.43

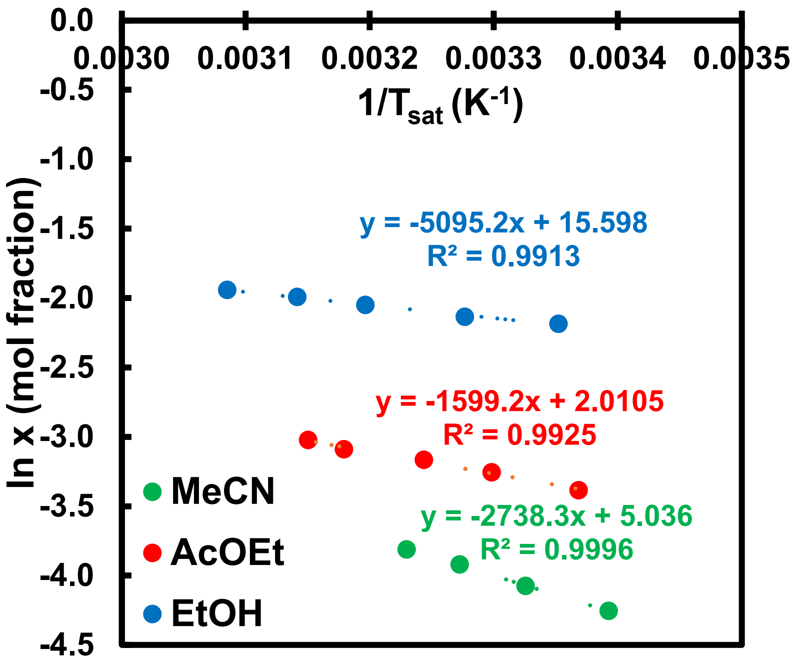

Figure S31: Van 't Hoff plots for 3-hydroxybenzoic acid.

Anthranilic acid (**17**)

Table S19: Anthranilic acid solubilities with Crystal16.

	Molar fraction x	Average T_{sat} (K)
EtOH	0.07297	287.58
	0.10451	305.45
	0.13092	316.45
	0.15430	324.62
MeCN	0.04539	299.92
	0.07768	314.08
	0.10263	322.68
	0.11930	328.95
AcOEt	0.05304	280.38
	0.06991	296.68
	0.08854	310.18
	0.10011	317.58

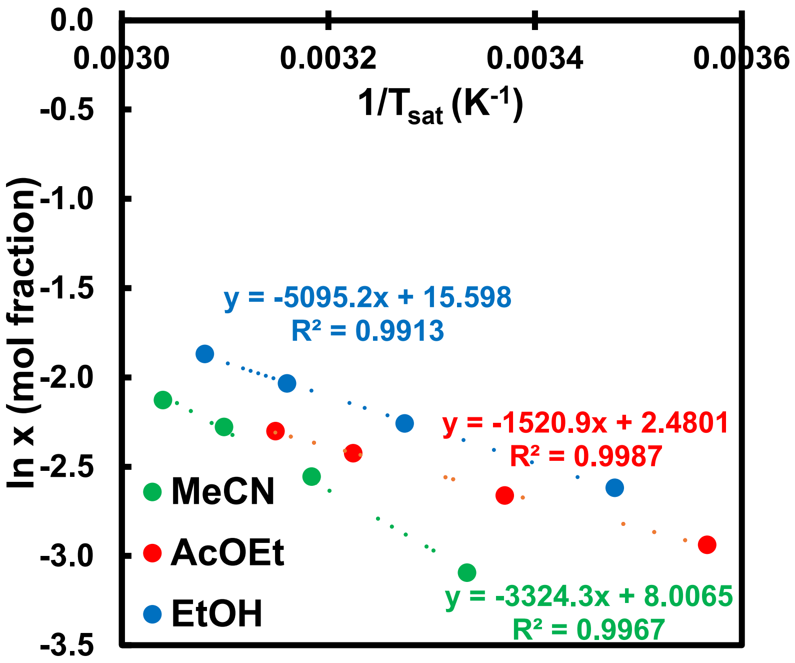

Figure S32: Van 't Hoff plots for anthranilic acid.

Vanillic acid (**20**)

Table S20: Vanillic acid solubilities with Crystal16.

	Molar fraction x	Average T_{sat} (K)
EtOH	0.03420	302.95
	0.04180	314.02
	0.04893	322.05
	0.03406	303.95
	0.03854	310.78
MeCN	0.00610	315.19
	0.00700	319.24
	0.00820	324.31
	0.00925	328.52
AcOEt	0.00628	298.91
	0.00753	307.94
	0.00843	314.34
	0.00935	318.97

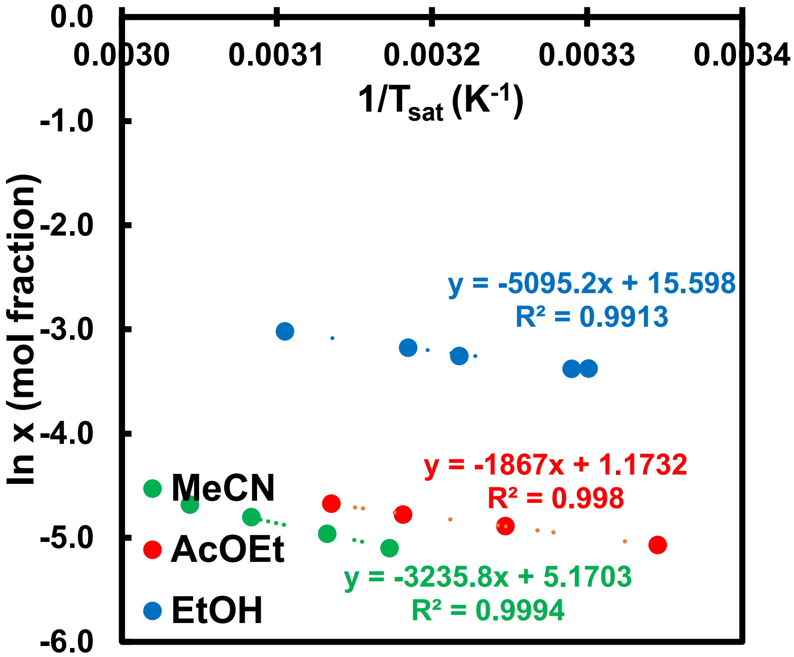

Figure S33: Van 't Hoff plots for vanillic acid.

4-nitrobenzoic acid (**21**)

Table S21: 4-nitrobenzoic acid solubilities with Crystal16.

	Molar fraction x	Average T_{sat} (K)
EtOH	0.00792	295.98
	0.01030	303.88
	0.01268	309.12
	0.01494	313.32
	0.01875	318.92
AcOEt	0.00508	301.62
	0.00596	307.45
	0.00731	315.28
	0.00859	321.82

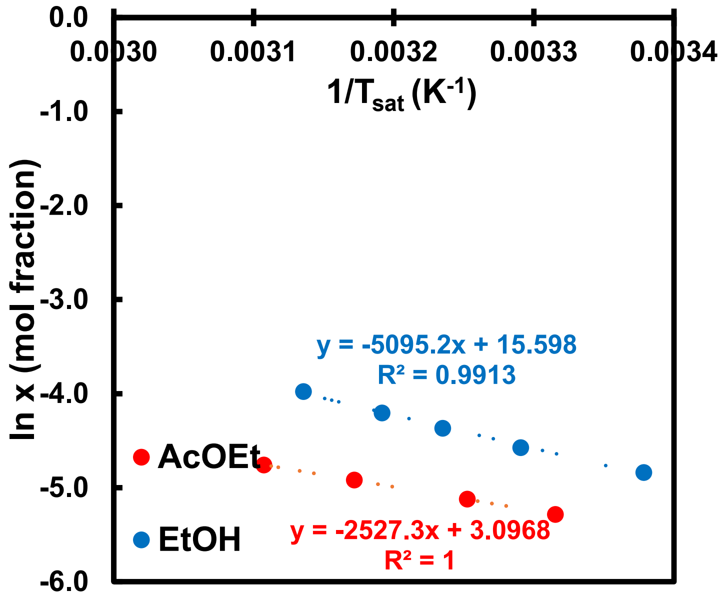

Figure S34: Van 't Hoff plots for 4-nitrobenzoic acid.

2,5-dihydroxybenzoic acid (**22**)

Table S22: 2,5-dihydroxybenzoic acid solubilities with Crystal16.

	Molar fraction x	Average T_{sat} (K)
EtOH	0.12645	284.88
	0.13976	297.55
	0.15176	316.65
	0.16106	324.15
	0.14059	309.35
	0.14593	314.55
	0.15436	321.35
	0.16044	325.75
MeCN	0.03037	309.98
	0.03766	319.38
	0.04502	327.85
	0.02700	305.62
	0.02940	308.92
	0.03065	310.85
	0.03573	317.88
AcOEt	0.05745	314.73
	0.06571	331.01
	0.05365	309.53
	0.05601	314.29
	0.05996	321.71
	0.06207	325.44

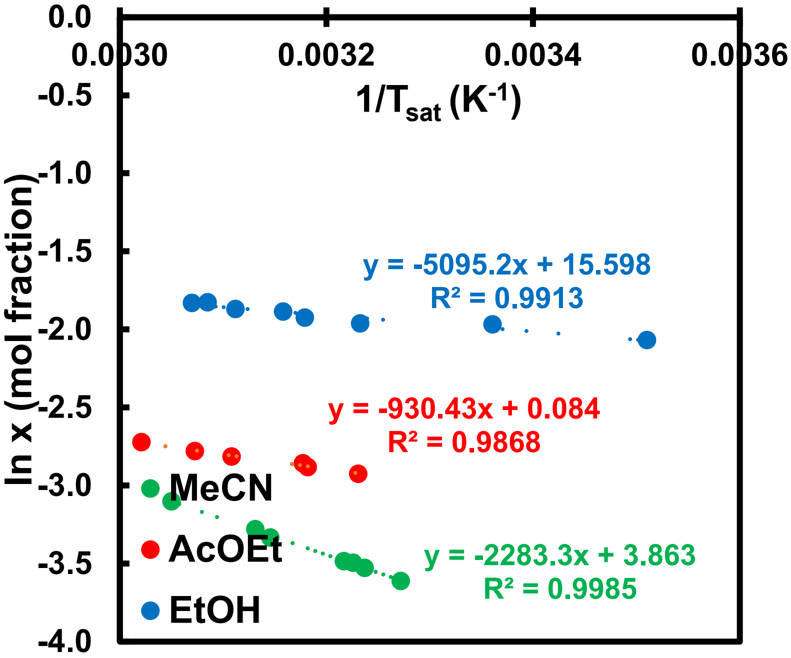

Figure S35: Van 't Hoff plots for 2,5-dihydroxybenzoic acid.

2-fluorobenzoic acid (**23**)

Table S23: 2-fluorobenzoic acid solubilities with Crystal16.

	Molar fraction x	Average T_{sat} (K)
**EtOH**	0.21026	297.98
	0.25579	310.25
	0.31040	323.98
	0.33159	326.42
**MeCN**	0.06421	294.32
	0.08018	300.75
	0.09646	306.18
	0.10800	309.72
**AcOEt**	0.13149	311.15
	0.14624	315.88
	0.15638	318.95
	0.16940	322.78
	0.09759	298.05
	0.11411	305.28

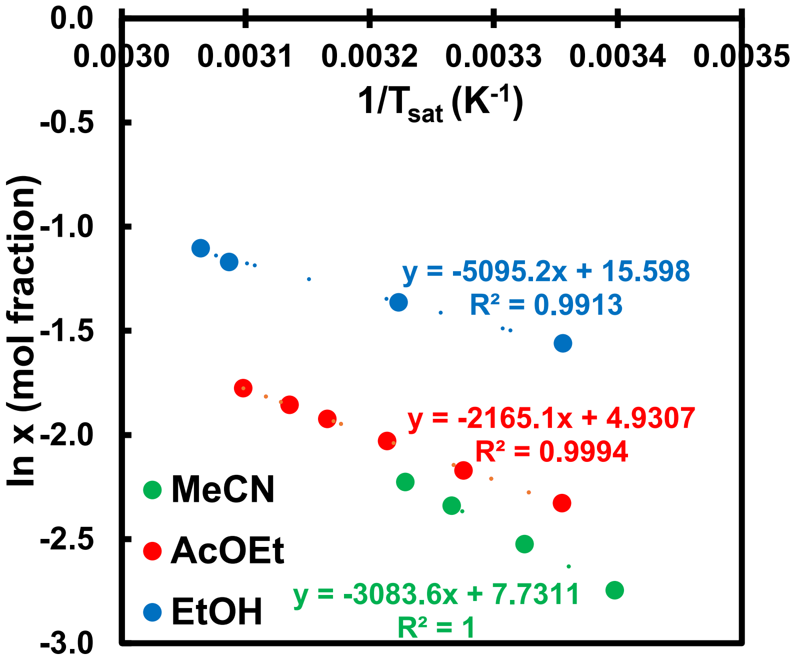

Figure S36: Van 't Hoff plots for 2-fluorobenzoic acid.

3,5-Dihydroxybenzoic acid (**24**)

Table S24: 3,5-dihydroxybenzoic acid solubilities with Crystal 16.

	Molar fraction x	Average T_{sat} (K)
MeCN	0.01455	309.75
	0.01843	316.45
	0.02408	323.98
	0.02860	328.58
	0.00669	287.05
	0.00914	296.88
	0.01194	303.65

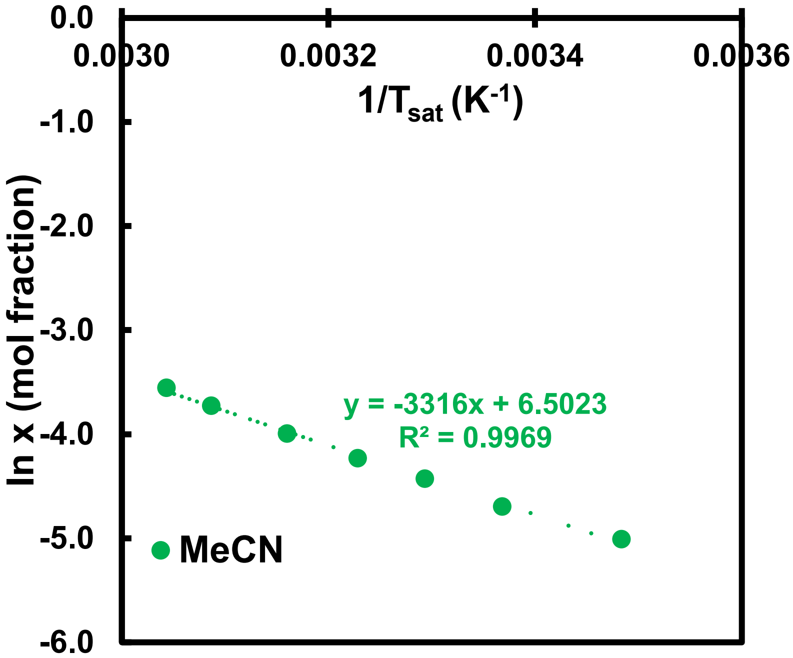

Figure S37: Van 't Hoff plots for 3,5-dihydroxybenzoic acid.

3-nitrobenzoic acid (**25**)

Table S25: 3-nitrobenzoic acid solubilities with Crystal16.

	Molar fraction x	Average T_{sat} (K)
MeCN	0.05277	298.95
	0.07554	308.78
	0.10244	314.15
	0.12569	320.85
	0.08768	313.45
	0.06196	303.42
	0.06965	306.58
AcOEt	0.08701	304.95
	0.09869	311.05
	0.12141	323.55

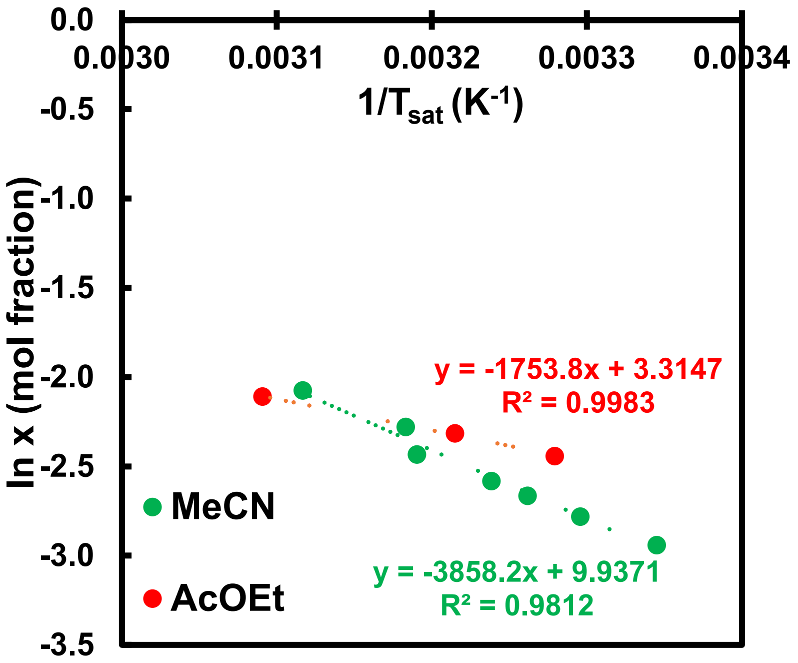

Figure S38: Van 't Hoff plots for 3-nitrobenzoic acid.

4-nitrophenol (**26**)

Table S26: 4-nitrophenol solubilities with Crystal16.

	Molar fraction x	Average T_{sat} (K)
EtOH	0.32995	296.05
	0.40186	321.60
	0.37231	312.38
	0.39650	320.38
AcOEt	0.27923	302.12
	0.29343	308.12
	0.31332	315.45
	0.33093	321.22

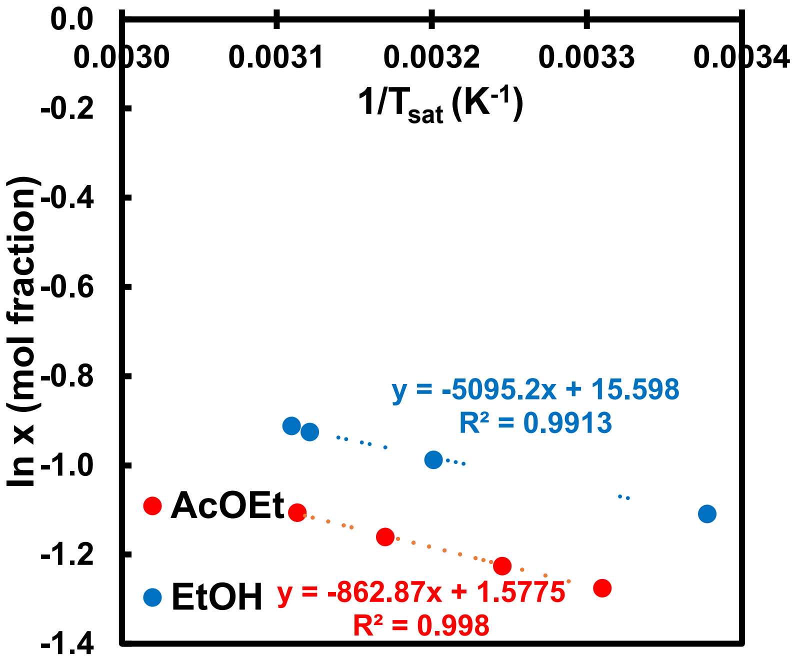

Figure S39: Van 't Hoff plots for 4-nitrophenol.

1-hydroxy-2-naphtoic acid (**27**)

Table S27: 1-hydroxy-2-naphtoic acid solubilities with Crystal 16.

	Molar fraction x	Average T_{sat} (K)
AcOEt	0.02221	315.28
	0.02592	322.95
	0.02895	331.95
	0.01765	302.88
	0.02010	309.25
	0.02069	311.38

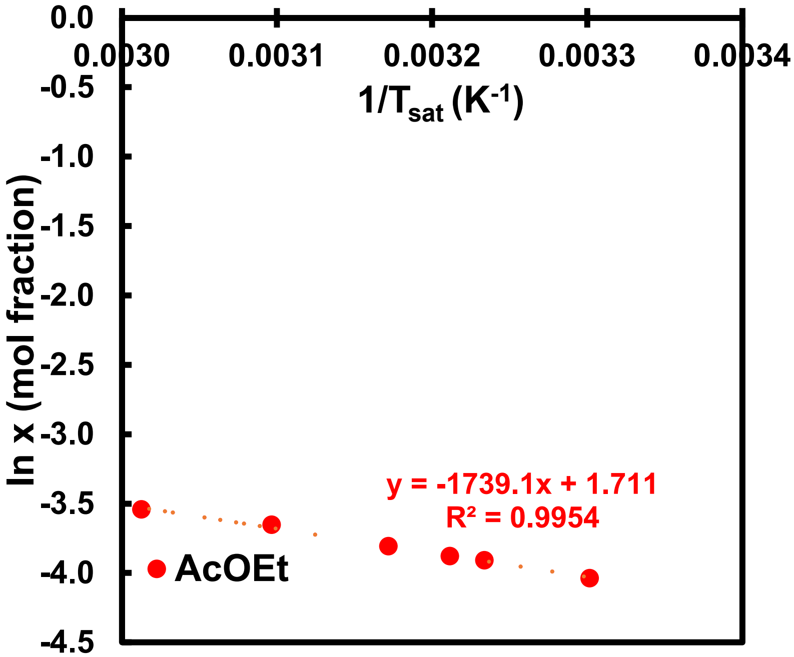

Figure S40: Van 't Hoff plots for 1-hydroxy-2-naphtoic acid.

2,4-dihydroxybenzoic acid (**28**)

Table S28: 2,4-dihydroxybenzoic acid solubilities with Crystal16.

	Molar fraction x	Average T_{sat} (K)
EtOH	0.11936	297.42
	0.14665	326.75
	0.12966	309.58
	0.14095	320.42
	0.15231	332.22
MeCN	0.03089	311.29
	0.03680	319.19
	0.04397	326.57
	0.04891	329.67
AcOEt	0.06524	328.34
	0.06844	330.90
	0.05774	308.44
	0.06127	318.69

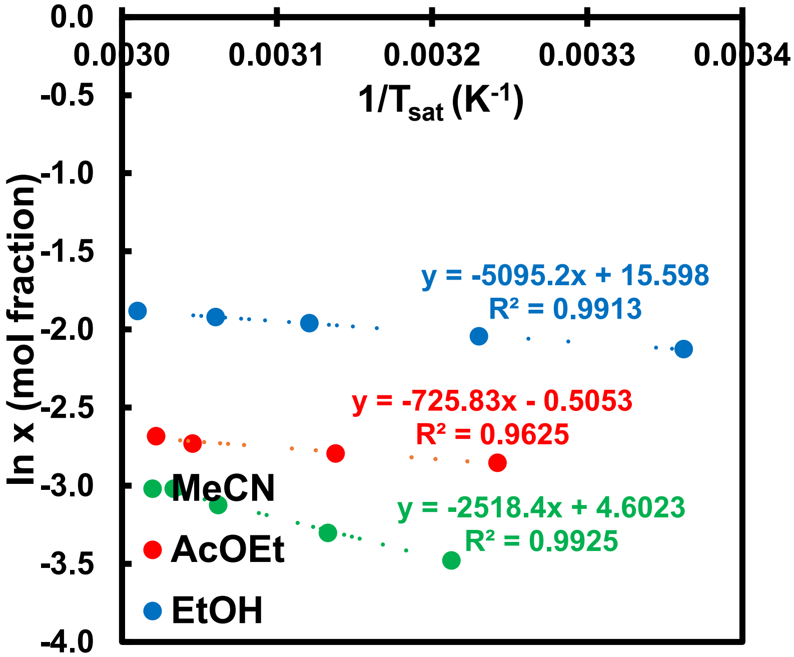

Figure S41: Van 't Hoff plots for 2,4-dihydroxybenzoic acid.

Orcinol (**29**)

Solubility measurements of orcinol in EtOH, MeCN and AcOEt were not possible as any mixture in these solvents lead to the formation of a viscous liquor for which crystallization never occurred when cooling. A possible explanation for this phenomenon is the relatively low melting point of that compound (109°C) and a suspected eutectic equilibrium whose temperature would be too low to induce supersaturation high enough when cooling.

Dodecanedioic acid (**30**)

Table S29: Dodecanedioic acid solubilities with Crystal 16.

	Molar fraction x	Average T_{sat} (K)
EtOH	0.00644	289.38
	0.00856	293.72
	0.00968	295.75
	0.01118	297.72
	0.01782	305.52
	0.02422	310.85
	0.03141	315.42

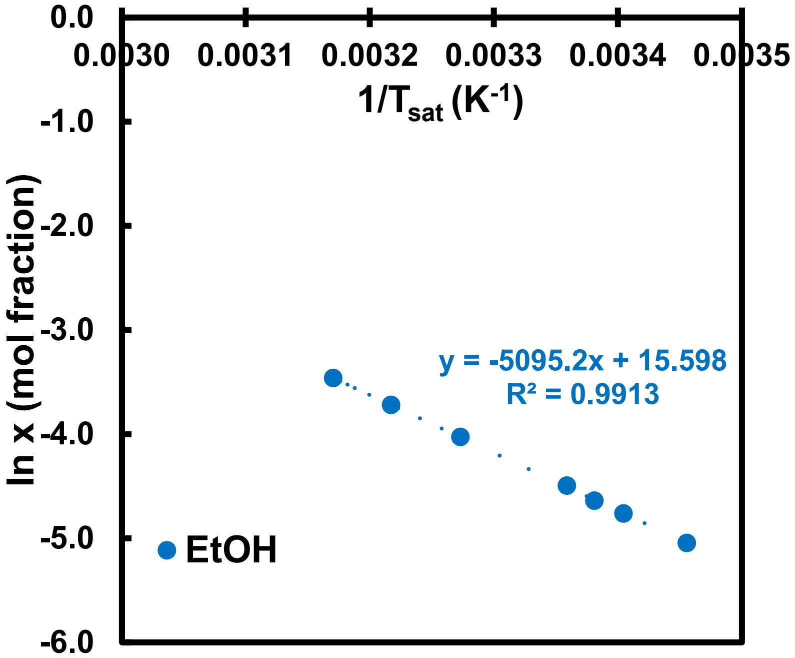

Figure S42: Van 't Hoff plots for dodecanedioic acid.

Table S30: Ternary compositions and saturation temperatures results screened by the STM method. 'T_r' is the highest value of theoretical saturation temperature computed from pure components Van 't Hoff plots and 'T_{sat}' is the average of three experimental measurements of saturation temperature. ([i]: positive hits for which screening was not performed in all three solvents as a cocrystal was found in one ; [ii]: false positive that could be due to overall solubility lowered by unfavourable interactions)

Rank	Coformer	Solvent	Concentration RS-PZQ (mol/L)	Concentration coformer (mol/L)	Ratio $M_{PZQ:cof}$ in solution (mol_{cof} per mol_{PZQ})	Theoretical T_r (K)	Experimental average T_{sat} (K)	ΔT (= $T_{sat} - T_r$)
1	Sebacic acid	EtOH	0.2891	0.4114	1.42	303.19	298.15	**-5.04**
		MeCN	Coformer insoluble					
		AcOEt	0.2082	0.0548	0.26	309.16	303.55	**-5.61**
2	Suberic acid	EtOH	0.2878	0.5995	2.08	302.56	296.72	**-5.84**
		MeCN	Coformer insoluble					
		AcOEt	Poor affinity between coformer and solvent					
3	Benzoic acid	EtOH	0.2866	3.8135	13.30	302.43	307.18	**4.75** [ii]
		MeCN	0.3255	1.0633	3.27	303.86	290.55	**-13.31**
		AcOEt	0.2129	2.7418	12.88	309.29	304.15	**-5.14**
4	Pimelic acid	EtOH	Solubility measurements not consistent					
		MeCN	0.3180	0.2384	0.75	303.65	289.52	**-14.13**
		AcOEt	0.2147	0.5055	2.36	309.11	302.58	**-6.53**
5	Salicylic acid	EtOH	0.2882	3.0522	10.59	302.53	291.15	**-11.38**
		MeCN	0.3259	0.7060	2.17	304.04	**Never crystallized upon cooling**	
		AcOEt	0.2077	1.9180	9.24	344.13	299.45	**-9.49**
6	1,4-Diiodotetrafluorobenzene[i]	EtOH	0.2856	1.4377	5.03	302.54	**Crystallized when mixed: never dissolved**	
7	4-Hydroxybenzoic acid[i]	EtOH	0.3203	2.6470	8.26	306.35	326.45	**20.1**
		MeCN	0.4089	0.3789	0.93	309.32	**Crystallized when mixed: never dissolved**	
8	Terephthalic acid	Coformer insoluble in all solvents tried: could not be screened with STM method						
9	4-Aminobenzoic acid	EtOH	0.2868	0.9609	3.35	302.64	280.58	**-22.06**
		MeCN	0.4091	0.5914	1.45	309.05	284.88	**-24.17**
		AcOEt	0.2062	0.6976	3.38	310.20	**Never crystallized upon cooling**	
10	Isophthalic acid	Coformer insoluble in all solvents tried: could not be screened with STM method						
11	Azelaic acid	EtOH	0.2895	1.1863	4.10	302.61	297.92	**-4.69**
		MeCN	Solubility measurements not consistent					
		AcOEt	0.2181	0.1644	0.75	309.53	298.52	**-11.01**
12	4-Aminosalicylic acid[i]	EtOH	Suspected degradation behaviour (solubility reducing over time)					
		MeCN	0.3237	0.1219	0.38	305.15	312.30	**7.15**
13	3,5-Dinitrobenzoic acid[i]	EtOH	System forming too dense crystals to be stirred					
		MeCN	0.3168	0.2834	0.89	303.75	326.72	**22.97**
14	*trans*-Cinnamic acid	EtOH	0.2949	1.5796	5.36	302.94	294.78	**-8.16**
		MeCN	0.3130	0.4353	1.39	303.68	288.72	**-14.97**
		AcOEt	0.2095	1.1415	5.45	308.74	302.05	**-6.69**

15	Hydroquinone[i]	EtOH	Degradation (solutions turning pink over time)					
		MeCN	0.3203	1.1689	3.65	303.54	317.75	14.21
16	3-Hydroxybenzoic acid	EtOH	0.2943 / 0.7009	2.2718 / 2.7447	7.72 / 3.92	303.02 / 319.97	Never crystallized upon cooling	
		MeCN	0.4110 / 0.6536	0.3862 / 0.5023	0.94 / 0.77	309.68 / 319.00	Never crystallized upon cooling	
		AcOEt	0.2073 / 0.2997	0.8648 / 1.0318	4.17 / 3.44	309.11 / 319.57	Never crystallized upon cooling	
17	Anthranilic acid	EtOH	0.3115	1.9452	6.25	303.93	Never crystallized upon cooling	
		MeCN	0.3150	0.9416	2.99	303.47	Never crystallized upon cooling	
		AcOEt	0.2158	1.8857	8.74	309.55	299.98	-9.57
18	Phthalic acid	Coformer insoluble in all solvents tried: could not be screened with STM method						
19	D-(-)-Tartaric acid	Solubility issues in all solvents tried						
20	Vanillic acid	EtOH	0.2913	0.5971	2.05	303.07	313.14	10.07
		MeCN	0.6628	0.1155	0.17	318.58	312.44	-6.14
		AcOEt	0.3057	0.1854	0.61	319.56	325.13	5.56
21	4-Nitrobenzoic acid	EtOH	0.2977	0.1783	0.60	303.21	300.41	-2.80
		MeCN	Coformer insoluble					
		AcOEt	0.2095	0.1223	0.58	309.00	Never crystallized upon cooling	
22	2,5-Dihydroxybenzoic acid	EtOH	0.6903	3.1355	4.54	320.84	275.05	-45.79
		MeCN	0.3220	0.4548	1.41	304.09	Crystallized when mixed: never dissolved	
		AcOEt	0.2987	1.2287	4.11	319.35	329.22	9.87
23	2-Fluorobenzoic acid	EtOH	0.2894	5.0500	17.45	302.76	298.28	-4.48
		MeCN	0.3130	1.6474	5.26	303.54	294.55	-8.99
		AcOEt	0.2182	2.7947	12.81	309.55	303.22	-6.33
24	3,5-Dihydroxybenzoic acid[i]	EtOH	Solubility measurements not consistent					
		MeCN	0.3079	0.2041	0.66	303.18	329.85	26.67
25	3-Nitrobenzoic acid	EtOH	Solubility measurements not consistent					
		MeCN	0.3119	1.1336	3.64	303.57	287.35	-16.22
		AcOEt	0.2117	2.0492	9.68	309.22	298.75	-10.47
26	4-Nitrophenol	EtOH	0.2685	8.6263	32.13	301.26	292.98	-8.28
		MeCN	Solubility too high and solubility measurements not consistent					
		AcOEt	0.2176	9.0790	41.65	316.36	312.55	-3.81
27	1-Hydroxy-2-naphtoic acid	EtOH	Suspected degradation (colour changes over time)					
		MeCN	Coformer insoluble					
		AcOEt	0.2147	0.1383	0.64	309.12	Never crystallized upon cooling	
28	2,4-Dihydroxybenzoic acid	EtOH	0.2899	2.3608	8.14	302.63	332.07	29.43
		MeCN	0.6625	0.6590	0.99	319.17	Crystallized when mixed: never dissolved	
		AcOEt	0.2166 / 0.3025	1.2401 / 1.3074	5.72 / 4.32	313.47 / 320.33	Crystallized when mixed: never dissolved	
29	Orcinol	Mixture of orcinol with any solvent forms of an undersaturated viscous liquor: no crystallization possible						
30	Dodecanedioic acid	EtOH	0.2920	0.2684	0.92	303.10	300.08	-3.02
		MeCN	Coformer insoluble					
		AcOEt	Poor affinity between coformer and solvent					
